# Verified iptables Firewall Analysis and Verification

**DOI:** 10.1007/s10817-017-9445-1

**Published:** 2018-01-03

**Authors:** Cornelius Diekmann, Lars Hupel, Julius Michaelis, Maximilian Haslbeck, Georg Carle

**Affiliations:** Department of Informatics, Technical University of Munich, Boltzmannstr. 3, Garching bei München, Germany

**Keywords:** Computer networks, Firewalls, Isabelle, Netfilter, Iptables, Semantics, Formal verification

## Abstract

This article summarizes our efforts around the formally verified static analysis of iptables rulesets using Isabelle/HOL. We build our work around a formal semantics of the behavior of iptables firewalls. This semantics is tailored to the specifics of the filter table and supports arbitrary match expressions, even new ones that may be added in the future. Around that, we organize a set of simplification procedures and their correctness proofs: we include procedures that can unfold calls to user-defined chains, simplify match expressions, and construct approximations removing unknown or unwanted match expressions. For analysis purposes, we describe a simplified model of firewalls that only supports a single list of rules with limited expressiveness. We provide and verify procedures that translate from the complex iptables language into this simple model. Based on that, we implement the verified generation of IP space partitions and minimal service matrices. An evaluation of our work on a large set of real-world firewall rulesets shows that our framework provides interesting results in many situations, and can both help and out-compete other static analysis frameworks found in related work.

## Introduction

Firewalls are a fundamental security mechanism for computer networks. Several firewall solutions, ranging from open source [66, 78, 79] to commercial [14, 37], exist. Operating and managing firewalls is challenging as rulesets are usually written manually. While vulnerabilities in firewall software itself are comparatively rare, it has been known for over a decade [82] that many firewalls enforce poorly written rulesets. However, the prevalent methodology for configuring firewalls has not changed. Consequently, studies regularly report insufficient quality of firewall rulesets [25, 36, 47, 54, 74, 81, 84–86].

**Fig. 1:**
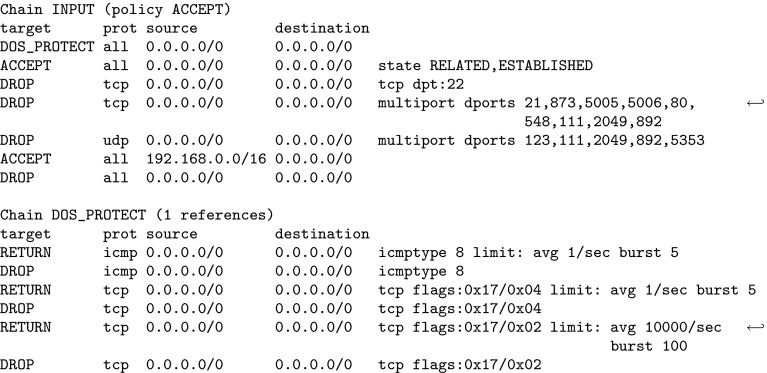
Linux iptables ruleset of a Synology NAS (network attached storage) device

The predominant firewall of Linux is iptables [78]. In general, an iptables ruleset is processed by the Linux kernel for each packet comparably to a batch program: rules are evaluated sequentially, but the action (sometimes called target) is only applied if the packet matches the criteria of the rule. A list of rules is called a chain. Ultimately, the Linux kernel needs to determine whether to ACCEPT or DROP the packet, hence, those are the common actions. Further possible actions include jumping to other chains and continue processing from there.

As an example, we use the firewall rules in Fig. 1, taken from an NAS (network-attached storage) device. The ruleset reads as follows: processing starts at the INPUT chain. In the first rule, all incoming packets are sent directly to the user-defined DOS_PROTECT chain, where some rate limiting is applied. A packet which does not exceed certain limits can make it through this chain without getting DROPped by RETURNing back to the second rule of the INPUT chain. In this second rule, the firewall allows all packets which belong to already ESTABLISHED (or RELATED) connections. This is generally considered good practice. Often, the ESTABLISHED rule accepts most packets and is placed at the beginning of a ruleset for performance reasons. However, it is barely interesting for the actual policy (“*who may connect to whom*”) enforced by the firewall. The interesting aspect is when a firewall accepts a packet which does not yet belong to an established connection. Once a packet is accepted, further packets for this connection are treated as ESTABLISHED. In the example, the subsequent rules are the interesting ones which shape the firewall’s connectivity policy. There, some services, identified by their ports, are blocked (and any packets with those destination ports will never create an established connection). Finally, the firewall allows all packets from the local network 192.168.0.0/16 and discards all other packets.

Several tools [47–49, 54, 59, 69, 80, 85] have been developed to ease firewall management and reveal configuration errors. Many tools are not designed for iptables directly, but are based on a generic firewall model. When we tried to analyze real-world iptables firewalls with the publicly available static analysis tools, none of them could handle the rulesets. Even after we simplified the firewall rulesets, we found that tools still fail to analyze our rulesets for the following reasons:

- They do not support the vast amount of firewall features,
- Their firewall model is too simplistic,
- They require the administrator to learn a complex query language which might be more complex than the firewall language itself,
- The analysis algorithms do not scale to large firewalls, or
- The output of the (unverified) verification tools itself cannot be trusted.

To illustrate the problem, we decided to use *ITVal* [48] because it natively supports iptables, is open source, and supports calls to user-defined chains. However, ITVal’s firewall model is representative of the model used by the majority of tools; therefore, the problems described here also apply to a large class of other tools. Firewall models used in related work are surveyed in Sect. 3.1.

We used ITVal to partition the IP space of Fig. 1 into equivalence classes (i.e., ranges with the same access rights) [49]. The expected result is a set of two IP ranges: the local network 192.168.0.0/16 and the “rest”. However, ITVal erroneously only reports one IP range: the universe. Removing the first two rules (in particular the call in the DOS_PROTECT chain) lets ITVal compute the expected result.

We identified two concrete issues which prevent tools from “understanding” real-world firewalls. First, calling and returning from custom chains, due to the possibility of complex nested chain calls. Second, more seriously, most tools do not understand the firewall’s match conditions. In the above example, the rate limiting is not understood. An ad-hoc implementation of rate limiting for the respective tool might not be possible, because the underlying algorithm might not be capable of dealing with this special case. Even so, this would not solve the general problem of unknown match conditions. Firewalls, such as iptables, support numerous match conditions and several new ones are added in every release. As of version 1.6.0 (Linux kernel 4.10, early 2017), iptables supports more than 60 match conditions with over 200 individual options. We expect even more match conditions for nftables [79] in the future since they can be written as simple userspace programs [45]. Therefore, it is virtually impossible to write a tool which understands all possible match conditions. Combined with the fact that in production networks, huge, complex, and legacy firewall rulesets have evolved over time, this poses a particular challenge. Our methodology to tackle this can also be applied to firewalls with simpler semantics, or younger technology with fewer features, e.g., Cisco IOS Access Lists or filtering OpenFlow flow tables (Sect. 15).

In this article, we first build a fundamental prerequisite to enable tool-supported analysis of *real-world* firewalls: we present several steps of semantics-preserving ruleset simplification, which lead to a ruleset that is “understandable” to subsequent analysis tools: first, we unfold all calls to and returns from user-defined chains. This process is exact and valid for arbitrary match conditions. Afterwards, we process unknown match conditions. For that, we embed a ternary-logic semantics into the firewall’s semantics. Due to ternary logic, all match conditions not understood by subsequent analysis tools can be treated as always yielding an unknown result. In a next step, all unknown conditions can be removed. This introduces an over- and underapproximation ruleset, called upper/lower closure. Guarantees about the original ruleset dropping/allowing a packet can be given by using the respective closure ruleset.

To summarize, we provide the following contributions for simplifying iptables rulesets:

1. A formal semantics of iptables packet filtering (Sect. 4)
2. Chain unfolding: transforming a ruleset in the complex chain model to a ruleset in the simple list model (Sect. 5)
3. An embedded semantics with ternary logic, supporting arbitrary match conditions, introducing a lower/upper closure of accepted packets (Sect. 6)
4. Normalization and translation of complex logical expressions to an iptables-compatible format, discovering a meta-logical firewall algebra (Sect. 7)

We give a small intermediate evaluation to demonstrate these generic ruleset preprocessing steps (Sect. 8). Afterwards, we use these preprocessing steps to build a fully-verified iptables analysis and verification tool on top. In detail, our further contributions are:

5. A simple firewall model, designed for mathematical beauty and ease of static analysis (Sect. 9)
6. A method to translate real-world firewall rulesets into this simple model (Sect. 10), featuring a series of translation steps to transform, rewrite, and normalize primitive match conditions (Sect. 11)
7. Static and automatic firewall analysis methods, based on the simple model (Sect. 12), featuring
  IP address space partitioning
  Minimal service matrices
8. Our stand-alone, administrator-friendly tool *fffuu* (Sect. 13)
9. Evaluation on large real-world data set (Sect. 14)
10. Full formal and machine-verifiable proof of correctness with Isabelle/HOL (Sect. 17)

## Background: Formal Verification with Isabelle

**Fig. 2:**
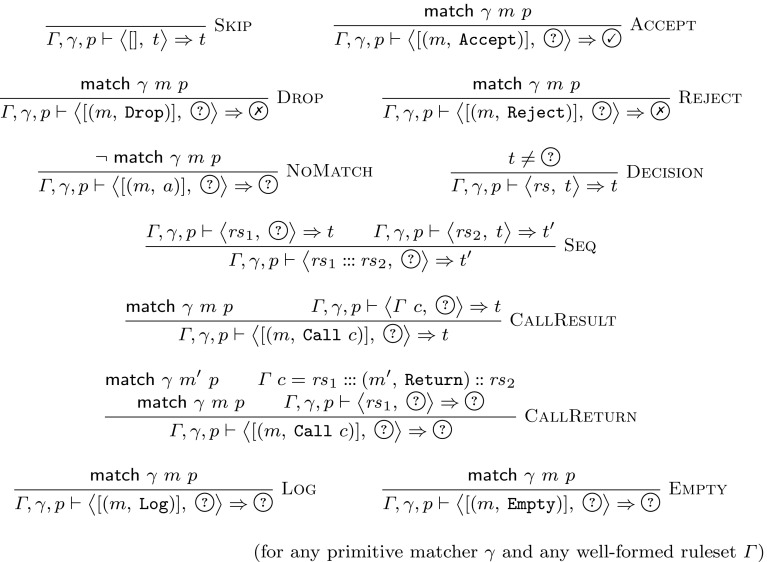
Big-step semantics for iptables

We verified all proofs with Isabelle [63], using its standard Higher-Order Logic (HOL). Isabelle is a proof assistant in the *LCF* tradition: the system is based on a small and well-established kernel. All higher-level specification and proof tools, e.g., for inductive predicates, functional programs, or proof search, have to go through this kernel. Therefore, the correctness of all obtained results only depends on the correctness of this kernel and the iptables semantics (Fig. 2).

The full formalization containing a set of Isabelle theory files is publicly available. An interested reader may consult the detailed (100+ pages) proof document. For brevity, we usually omit proofs in this article, but point the reader with a footnote to the corresponding part of the formalization. Section 17 points the reader to our Isabelle formalization and further accompanying material.

*Notation.* We use pseudo code close to SML and Isabelle. Function application is written without parentheses, e.g., $f\ a$ denotes function *f* applied to parameter *a*. We write  for prepending a single element to a list, e.g., , and  for appending lists, e.g., . The empty list is written as []. $[f\ a.\ a \leftarrow l]$ denotes a list comprehension, i.e., applying *f* to every element *a* of list *l*. $[f\ x\ y.\ \ x \leftarrow l_1,\; y \leftarrow l_2]$ denotes the list comprehension where *f* is applied to each combination of elements of the lists $l_1$ and $l_2$. For $f\ x \ y = (x,\,y)$, this yields the Cartesian product of $l_1$ and $l_2$.

Whenever we refer to specific iptables options or modules, we set them in typewriter font. The iptables options can be looked up in the respective man pages iptables(8) and iptables-extensions(8).

## Related Work

We first survey the common understanding of firewalls in the literature and present specific static firewall analysis tools afterwards.

### Firewall Models

Packets are routed through the firewall and the firewall needs to decide whether to allow or deny a packet. The firewall’s ruleset determines its filtering behavior. The firewall inspects its ruleset for each single packet to determine the action to apply to the packet. The ruleset can be viewed as a list of rules; usually it is processed sequentially and the first matching rule is applied.

The literature agrees on the definition of a single firewall rule. It consists of a predicate (the match expression) and an action. If the match expression applies to a packet, the action is performed. Usually, a packet is scrutinized by several rules. Zhang et al. [86] specify a common format for packet filtering rules. The action is either “allow” or “deny”, which directly corresponds to the firewall’s filtering decision. The ruleset is processed strictly sequentially, no jumping between chains is possible. Yuan et al. [85] call this the *simple list model*. ITVal also supports calls to user-defined chains as an action. This allows “jumping” within the ruleset without having a final filtering decision yet. This is called the *complex chain model* [85].

In general, a packet header is a bitstring which can be matched against [87]. Zhang et al. [86] support matching on the following packet header fields: IP source and destination address, protocol, and port on layer 4. This model is commonly found in the literature [6, 9, 10, 69, 85, 86]. ITVal extends these match conditions with flags (e.g., TCP SYN) and connection states (INVALID, NEW, ESTABLISHED, RELATED). The state matching is treated as just another match condition.^1^ This model is similar to Margrave’s model for IOS [54]. When comparing these features to the simple firewall in Fig. 1, it becomes obvious that none of these tools supports that firewall directly.

We are not the first to propose simplifying firewall rulesets to enable subsequent analysis. Brucker et al. [8, 10, 11] provide algorithms to generate test cases from a firewall policy. A firewall policy in their model is a list of rules on disjoint networks. A rule is a partial function from packets to decisions, e.g., allow or deny. To keep the number of test cases manageable, the firewall ruleset is first simplified while preserving the original behavior. The correctness of these transformations is proved with Isabelle/HOL. With regard to low-level firewall features, the instantiation used by Brucker et al. in their evaluation is more limited than the model used by the tools presented above. This is not a limitation since their framework is designed to support different firewall technologies by having a more abstract and generic policy model. Yet, it demonstrates that our tool as a preprocessor to transform low-level iptables rules to a generic firewall model is a useful building block. In general, using our tool as preprocessor can make firewall analysis tools from related work available for iptables.

We are not aware of any tool which uses a model fundamentally different than those described here. Our model enhances existing work in that we use ternary logic to support arbitrary match conditions. To analyze a large iptables firewall, the authors of Margrave [54] translated it to basic Cisco IOS access lists [14] by hand. With our simplification, we can automatically remove all features not understood by basic Cisco IOS. This enables translation of any iptables firewall to basic Cisco access lists which is guaranteed to drop no more packets than the original iptables firewall. This opens up all tools available only for Cisco IOS access lists, e.g., Margrave [54] and Header Space Analysis [41].^2^

### Static Firewall Analysis Tools

Popular tools for static firewall analysis include FIREMAN [85], Capretta et al. [13], and the Firewall Policy Advisor [2]. They can use the following features to match on packets: IP addresses, ports, and protocol. However, most real-world firewall rulesets we found in our evaluation use many more features. As can be seen in Fig. 1, among others, iptables supports matching on source IP address, layer 4 port, inbound interface, conntrack state, entries and limits in the recent list. Hence, these tools would not be applicable (without our generic preprocessing) to most firewalls from our evaluation.

Most aforementioned tools allow detecting conflicts between rules to uncover configuration mistakes. Since our approach rewrites rules to a simpler form and the provenance and relation of the simplified rules to the original ruleset is lost, our approach does not support this. However, we offer service matrices (Sect. 12.2) to provide a general overview of the firewall’s filtering behavior.

The work most similar to our static analysis tool, in particular to our IP address space partitioning, is ITVal [48]: it supports a large set of iptables features and can compute an IP address space partition [49]. ITVal, as an academic prototype, only supports IPv4, is not formally verified, and its implementation contains several errors. For example, ITVal produces spurious results if the number of significant bits in IP addresses in CIDR notation [31] is not a multiple of 8. It does not consider logical negations which may occur when ${\mathtt {RETURN}}$ing prematurely from user-defined chains, which leads to wrong interpretation of complement sets. It does not support abstracting over unknown match conditions but simply ignores them, which also leads to spurious results. Anecdotally, we uncovered these corner cases when we tried to prove the correctness of our algorithms and Isabelle was presenting unexpected proof obligations. Without the formal verification, our tool would likely contain similar errors. For rulesets with more than 1000 rules, ITVal requires tens of GBs of memory. We are uncertain whether this is a limitation of its internal data structure or just a simple memory leak. ITVal neither proves the soundness nor the minimality of its IP address range partitioning. Nevertheless, ITVal shows the need for and the use of IP address range partitioning and has demonstrated that its implementation works well on rulesets which do not trigger the aforementioned errors. Our tool strongly builds on the ideas of ITVal, but with a different algorithm.

Exodus [57] translates existing device configurations to a simpler model, similar to our translation step. It translates router configurations to a high-level SDN controller program, which is implemented on top of OpenFlow. Exodus supports many Cisco IOS features. The translation problem solved by Exodus is comparable to this article’s problem of translating to a simple firewall model: OpenFlow 1.0 only supports a limited set of features (comparable to our simple firewall) whereas IOS supports a wide range of features (comparable to iptables). A complex language is ultimately translated to a simple language, which is the ‘hard’ direction.

Since our approach loses the relation of the simplified rules to the original ruleset, our approach cannot point to individual flawed firewall rules, but only provides a complete overview. For example, our tool reduces thousands of firewall rules to the easy-to-understand graph in Fig. 8, but the information which initial firewall rules and match conditions are responsible for each edge of the graph is lost. Complementary to our verification tool, and well-suited for debugging and uncovering responsible misbehaving rules, is Margrave [54]. Margrave can be used to query firewalls and to troubleshoot configurations or to show the impact of ruleset edits. Margrave can find scenarios, i.e., it can show concrete packets which violate a security policy. Our framework does not show such information. Margrave’s query language, which a potential user has to learn, is based on first-order logic.

All these tools have one limitation in common: they do not understand all iptables match conditions. Our generic ruleset preprocessing algorithms help to make a ruleset accessible for the respective tool. However, our generic algorithms still lose too much information. This is because iptables conditions are also related to each other. For example, the iprange module allows to write down IP address ranges using a notation more expressive than most tools support. Just removing iprange matches would lose too much information, since tools understand matches on IP address ranges in a simpler format. We need to rewrite iprange expressions to a simpler, semantics-preserving notation of IP addresses, commonly understood by tools. This may be non-trivial since one rule with one iprange expression may correspond to several rules with only simple matches on IP addresses. As a more involved example, we saw that most firewall analysis tools do not support matching on interfaces. But given that a firewall implements spoofing protection and the routing tables are known, conditions matching on network interfaces can be rewritten to those matching on IP addresses. After an intermediate evaluation (Sect. 8), we present in Sect. 11 algorithms to overcome these issues for the most common match conditions.

## Semantics of iptables

We formalized the semantics of a subset of iptables. The semantics focuses on access control, which is done in the INPUT, OUTUT, and FORWARD chain of the filter table. Thus packet modification (e.g., NAT) is not considered (and also not allowed in these chains).

Match conditions, e.g., source 192.168.0.0/24 and protocol TCP, are called *primitives*. A primitive matcher $\gamma$ decides whether a packet matches a primitive. Formally, based on a set *X* of primitives and a set of packets *P*, a primitive matcher $\gamma$ is a binary relation over *X* and *P*. The semantics supports arbitrary packet models and match conditions, hence both remain abstract in our definition.

In one firewall rule, several primitives can be specified. Their logical connective is conjunction, for example $\texttt {src 192.168.0.0/24}$ and ${\texttt {tcp}}$. Disjunction is omitted because it is neither needed for the formalization nor supported by the iptables user interface; this is consistent with the model by Jeffrey and Samak [39]. Primitives can be combined in an algebra of *match expressions* $M_X$: 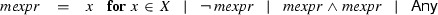


The match expression ${\mathsf {Any}}{}$ matches any packet. For a primitive matcher $\gamma$ and a match expression $m \in M_X$, we write ${{\mathsf {match}\ {\gamma }}\ m \ p}$ if a packet $p \in P$ matches *m*, essentially lifting $\gamma$ to a relation over $M_X$ and *P*, with the connectives defined as usual. With completely generic *P*, *X*, and $\gamma$, the semantics can be considered to have access to an oracle which understands all possible match conditions.

Furthermore, we support the following *actions*, modeled closely after iptables: ${\mathtt {Accept}}$, ${\mathtt {Reject}}$, ${\mathtt {Drop}}$, ${\mathtt {Log}}$, ${\mathtt {Empty}}$, ${\mathtt {Call}}\ c$ for a chain *c* , and ${\mathtt {Return}}$. A *rule* can be defined as a tuple $(m,\,a)$ for a match expression *m* and an action *a*. A list (or sequence) of rules is called a *chain*. For example, the beginning of the ${\texttt {DOS\_PROTECT}}$ chain in Fig. 1 is $[({\texttt {icmp}} \wedge {\texttt {icmptype 8 limit:}} \,\dots ,\, {\mathtt {Return}}),\, \dots ]$.

A set of named chains is called a *ruleset*. Let $\varGamma$ denote the mapping from chain names to chains. For example, $\varGamma \ {\texttt {DOS\_PROTECT}}$ returns the contents of the ${\texttt {DOS\_PROTECT}}$ chain. We assume that $\varGamma$ is well-formed, that is, if a ${\mathtt {Call}}\ c$ action occurs in a ruleset, then the chain named *c* is defined in $\varGamma$. This assumption is justified, because the Linux kernel only accepts well-formed rulesets.

### Inductive Definition

The semantics of a firewall wrt a given packet *p*, a background ruleset $\varGamma$, and a primitive matcher $\gamma$ can be defined as a relation over the currently active chain and the state before and the state after processing this chain. The semantics is specified in Fig. 2.^3^ The judgement ${\varGamma ,\gamma ,p \vdash \big \langle rs ,\; t \big \rangle \Rightarrow t'}$ states that starting with state *t*, after processing the chain $rs$, the resulting state is $t'$. For a packet *p*, our semantics focuses on firewall filtering decisions. Therefore, only the following three states are necessary: the firewall may allow ($\textcircled {\checkmark }$) or deny () the packet, or it may not have come to a decision yet ($\textcircled {?}$).

We will now discuss the most important rules.

$$\textsc {Accept}$$
If the packet *p* matches the match expression *m*, then the firewall with no filtering decision ($\textcircled {?}$) processes the singleton chain $[(m,\,{\mathtt {Accept}})]$ by switching to the allow state.
$$\textsc {Drop}/\textsc {Reject}$$
Both actions deny a packet. The difference lies in whether the firewall generates some informational message, which does not influence filtering.
$$\textsc {NoMatch}$$
If the firewall has not come to a filtering decision yet it can process any non-matching rule without changing its state.
$$\textsc {Decision}$$
As soon as the firewall made a filtering decision, all remaining rules can be skipped. Given determinism (Theorem 2), this means that once decided, the firewall does not change its filtering decision of $\textcircled {\checkmark }$ or .
$$\textsc {Seq}$$
If the firewall has not come to a filtering decision and it processes the chain $$ rs _1$$, which results in state *t* and starting from *t* processes the chain $$ rs _2$$, which results in state $$t'$$, then both chains can be processed sequentially, ending in state $$t'$$.
$$\textsc {CallResult}$$
If a matching ${\mathtt {Call}}$ to a chain named “*c*” occurs, the resulting state *t* is the result of processing the chain $\varGamma \; c$.
$$\textsc {CallReturn}$$
Likewise, if processing a prefix $$ rs _1$$ of the called chain does not lead to a filtering decision and directly afterwards, a matching $${\mathtt {Return}}$$ rule occurs, the called chain is processed without result.
$$\textsc {Log}/\textsc {Empty}$$
Neither rule influences the filtering behavior. An Empty rule, i.e., a rule without an action, is sometimes used by administrators to have iptables only update its internal state, e.g., updating packet counters.

**Fig. 3:**
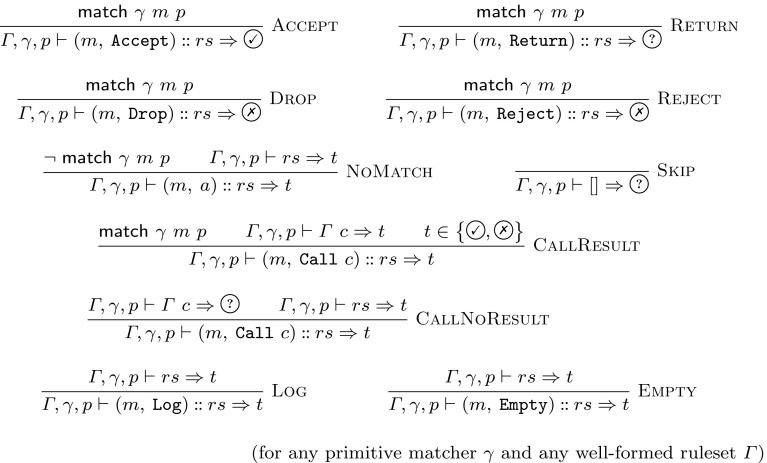
Alternative big-step semantics for iptables

The semantics is carefully designed to not require a call stack. The format of the CallReturn rule is part of this design: if we tried to introduce a rule that allows to process a ${\mathtt {Return}}$ without either processing its matching ${\mathtt {Call}}$ or manipulating some call stack, we would necessarily cause problems with the Seq rule. This is because a separated rule for ${\mathtt {Return}}$ would need to remain in the $\textcircled {?}$ state, and a later rule from the same chain (where we should already have returned from) could then switch to a decision state. One way of avoiding this problem is to merge the functionality of the Seq and Decision rules into all other rules. After doing so, one can introduce a separate Return rule and additionally remove the initial state, since it would always be $\textcircled {?}$. An example set of productions for such an alternate formulation is shown in Fig. 3. For the practical implementation of our proofs, this alternative lacks flexibility: since the Seq rule is no longer applicable, we cannot easily separate arguments about lists of rules from arguments about the different action types of rules. We provide this as an equivalent^4^ alternative because we hope that can provide additional confidence in the correctness of our semantics:

#### Theorem 1

(Equivalence of the two Semantics) If no call to *c* occurs in any of the chains of $\varGamma$ and  is either ${\mathtt {Accept}}$ or ${\mathtt {Drop}}$, then
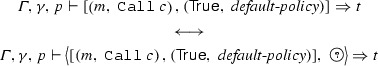


Note that for finite rulesets (i.e., the image/range of $\varGamma$ is finite), we can always find a *c* such that no call occurs to it. In practice, we will chose *c* to be INPUT, FORWARD, or OUTPUT. The Linux kernel rejects rulesets where a user calls these chains directly.

### Model Limitations and Stateful Matchers

Our primitive matcher is completely stateless: $\gamma :{:} \left( X \Rightarrow P \Rightarrow \mathbb {B}\right)$. However, iptables also allows stateful operations, such as marking a packet, and, later on, matching on the marking.

The documentation of iptables distinguishes between *match extensions* and *target extensions*. Ideally, almost all match extensions can be used as if they were stateless. Anything which performs an action should be implemented as target extension, i.e., action. For example, marking a packet with CONNMARK is an action. Matching on a CONNMARK marking is a match condition. Our semantics does not support the CONNMARK action. This is not a problem since usually, new CONNMARK markings are not set in the filter table. However, it is possible to match on existing markings. Since our primitive matchers and packets are completely generic, this case can be represented within our semantics: instead of keeping an internal CONNMARK state, an additional “ghost field” must be introduced in the packet model. Since packets are immutable, this field cannot be set by a rule, but the packet must be given to the firewall with the final value of the ghost field already set. Hence, an analysis must be carried out with the correct value in the ghost fields when the packet is given to the filter table. We admit that this model is very unwieldy in general. However, for one of the most used stateful modules of iptables, namely connection state tracking with conntrack and state, this model has been proven to be very convenient.^5^ We will elaborate on stateful connection tracking (which can be fully supported by our semantics) in Sect. 11.2. For future work, if we want to consider e.g., the raw or mangle table with its extended set of actions or OpenFlow with its full set of actions, a semantics needs to be designed with a mutable packet model.

What if a match extension maintains an internal state and changes its behavior on every invocation? Ideally, due to usability, iptables match extensions should be “purely functional”; however, the recent and connbytes modules exhibit side effects on their internal state. As a consequence, the tautology in Boolean logic “$a \wedge \lnot a = \mathsf {False}$” does not hold if *a* is a module which updates an internal state and its matching behavior after every invocation. Therefore, one might argue that our iptables model can only be applied to stateless match conditions. If we add some state $\sigma$ and updated state $\sigma '$ to the match condition, the formula “$a_\sigma \wedge \lnot a_{\sigma '}$” now correctly represents stateful match conditions. Therefore, it would only be wrong to perform equality operations on stateful match conditions, but not to model stateful match conditions with a specific fixed state. To additionally convince the reader of the soundness of our approach, it would be possible to adapt the parser to give a unique identifier to every primitive which is not known to be stateless. This identifier represents the internal state of that particular match condition at that particular position in a ruleset. It prevents equality operations between multiple invocations of a stateful match condition. This does not change any of our algorithms.

### Analysis and Use of the Semantics

The subsequent sections of this article are all based on these semantics. Whenever we provide a procedure $\mathsf {P}$ to operate on chains, we proved that the firewall’s filtering behavior is preserved, formally: 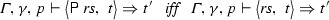 All our proofs are machine-verified with Isabelle. Therefore, once the reader is convinced of the semantics as specified in Fig. 2, the correctness of all subsequent theorems follows automatically, without any hidden assumptions or limitations.

The rules in Fig. 2 are designed such that every rule can be inspected individually. However, considering all of them together, it is not immediately clear whether the result depends on the order of their application to a concrete ruleset and packet. Theorem 2 states that the semantics is deterministic, i.e., only one uniquely defined outcome is possible.^6^

#### Theorem 2

(Determinism) 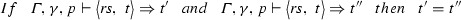


Next, we show that the semantics are actually total, i.e., there is always a decision for any packet and ruleset.^7^ We assume that the ruleset does not have an infinite loop and that all chains which are called exist in the background ruleset. These conditions are checked by the Linux kernel and can thus safely be assumed. In addition, we assume that only the actions defined in Fig. 2 occur in the ruleset; our parser rejects everything else.

We start any analysis with , where the default policy is either ${\mathtt {Accept}}$ or ${\mathtt {Drop}}$. This means that existing top-level ${\mathtt {Return}}$ actions fall back to the default policy. This is consistent with iptables behavior.

#### Theorem 3

(Totality) If the caller–callee relation is well-founded (i.e., there are no infinite calling loops), $\varGamma$ is well-formed (i.e., all chain names that are called are defined), there is no ${\mathtt {Return}}$ on top-level, and all actions are supported by the semantics, then 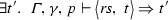


To also assert empirically that we only allow analysis of iptables rulesets which are total according to our semantics, we always check the preconditions of Theorem 3 at runtime when our tool loads a ruleset: first, we can statically verify that $\varGamma$ is well-formed by verifying that all chain names which are referenced in an action are defined and that no unsupported actions occur. Next, our tool verifies that there are no infinite loops by unfolding the ruleset (Sect. 5) only a finite but sufficiently large number of times and aborts if the ruleset is not in the proper form afterwards. These conditions have only been violated for a negligible fraction of all real-world firewalls we have analyzed. Those used very special iptables actions^8^ not supported by our semantics or special hand-crafted firewalls which deliberately violate a property and which are also rejected by the Linux kernel.

## Custom Chain Unfolding

In this section, we present algorithms to convert a ruleset from the complex chain model to the simple list model.

The function $\mathsf {pr}$ (“process return”) iterates over a chain. If a ${\mathtt {Return}}$ rule is encountered, all subsequent rules are amended by adding the ${\mathtt {Return}}$ rule’s negated match expression as a conjunct. Intuitively, if a ${\mathtt {Return}}$ rule occurs in a chain, all following rules of this chain can only be reached if the ${\mathtt {Return}}$ rule does not match. 
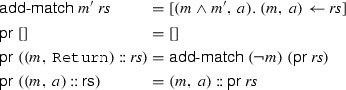


The function $\mathsf {pc}$ (“process call”) iterates over a chain, unfolding one level of ${\mathtt {Call}}$ rules. If a ${\mathtt {Call}}$ to the chain *c* occurs, the chain itself (i.e., $\varGamma \ c$) is inserted instead of the ${\mathtt {Call}}$. However, ${\mathtt {Return}}$s in the chain need to be processed and the match expression for the original ${\mathtt {Call}}$ needs to be added to the inserted chain. 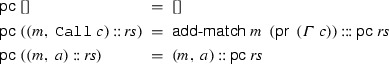


The procedure $\mathsf {pc}$ can be applied arbitrarily many times and preserves the semantics.^9^

### Theorem 4

($\mathsf {pc}$ sound and complete) 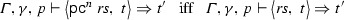


In each iteration, the algorithm unfolds one level of ${\mathtt {Call}}$s. The algorithm needs to be applied until the result no longer changes. Note that the syntax and semantics allow non-terminating rulesets. However, the only rulesets that are interesting for analysis are the ones actually accepted by the Linux kernel. Since it rejects rulesets with loops,^10^ both our algorithm and the resulting ruleset are guaranteed to terminate.

### Corollary 1

Every ruleset (with only ${\mathtt {Accept}}$, ${\mathtt {Drop}}$, ${\mathtt {Reject}}$, ${\mathtt {Log}}$, ${\mathtt {Empty}}$, ${\mathtt {Call}}$, ${\mathtt {Return}}$ actions) accepted by the Linux kernel can be unfolded completely while preserving its filtering behavior.

Since we have not formally verified the Linux kernel sources, Corollary 1 is not formally proven. It follows from our previous theorems and we have extensively checked it empirically.

In addition to unfolding calls, the following transformations applied to any ruleset preserve the semantics:

- replacing ${\mathtt {Reject}}$ actions with ${\mathtt {Drop}}$ actions,^11^
- removing ${\mathtt {Empty}}$ and ${\mathtt {Log}}$ rules,^12^
- simplifying match expressions which contain ${\mathsf {Any}}{}$ or $\lnot \,{\mathsf {Any}}{}$,^13^
- for some given primitive matcher, specific optimizations,^14^ e.g., rewriting src 0.0.0.0/0 to ${\mathsf {Any}}{}$.

**Fig. 4:**
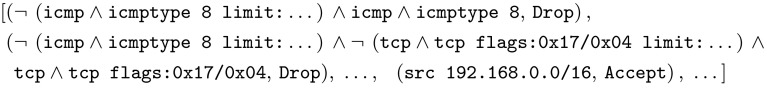
Unfolded Synology firewall

Therefore, after unfolding and optimizing, a chain which only contains ${\mathtt {Accept}}$ or ${\mathtt {Drop}}$ actions is left. In the subsequent sections, we require this as a precondition. As an example, recall the firewall in Fig. 1. Its ${\texttt { INPUT }}$ chain after unfolding and optimizing is listed in Fig. 4. Observe that some of the computed match expressions are beyond the expressiveness of what the iptables command line user interface supports. We will elaborate on this in Sect. 7.

## Unknown Primitives

As we argued earlier, it is infeasible to support all possible primitives of a firewall. Suppose a new firewall module is created which provides the ${\texttt { ssh\_blacklisted }}$ and ssh_innocent primitives. The former applies if an IP address has had too many invalid SSH login attempts in the past; the latter is the opposite of the former. Since we invented these primitives, no existing tool will support them. However, a new version of iptables could implement them or they may be provided as third-party kernel modules. Therefore, our ruleset transformations must take unknown primitives into account. To achieve this, we lift the primitive matcher $\gamma$ to ternary logic, adding $\mathsf {Unknown}$ as matching outcome. We embed this new “approximate” semantics into the semantics described in the previous sections. Thus, it becomes easier to construct matchers tailored to the primitives supported by a particular tool.

### Ternary Matching

Logical conjunction and negation on ternary values are as in Boolean logic, with these additional rules for $\mathsf {Unknown}$ operands (commutative cases omitted): 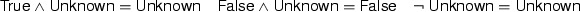 These rules correspond to Kleene’s 3-valued logic [42] and are well-suited for firewall semantics. For firewall rules, the first equation states that, if one condition matches, the final result only depends on the other condition. The next equation states that a rule cannot match if one of its conditions does not match. Finally, by negating an unknown value, no additional information can be inferred. The match expression ${\mathsf {Any}}{}$ always evaluates to $\mathsf {True}$ and $\lnot \;{\mathsf {Any}}{}$ always evaluates to $\mathsf {False}$ for any $\gamma$. A match expression may evaluate to $\mathsf {Unknown}$ if it contains unknown primitives $x \in X$.

We demonstrate the $\lnot \, \mathsf {Unknown} = \mathsf {Unknown}$ case by example: the two rulesets $\left[ ({\texttt {ssh\_blacklisted}},\, {\mathtt {Drop}})\right]$ and $\left[ ({\mathsf {Any}}{},\, {\mathtt {Call}}\ c)\right]$ where $\varGamma \, c\, = [(\mathtt {ssh\_innocent},\, {\mathtt {Return}}),\, ({\mathsf {Any}}{},\, {\mathtt {Drop}})]$ have exactly the same filtering behavior. After unfolding, the second ruleset collapses to $\left[ (\lnot \; {\texttt {ssh\_innocent}},\, {\mathtt {Drop}})\right]$. Both the ssh_blacklisted and the ssh_innocent primitives are $\mathsf {Unknown}$ to our matcher. Thus, since both rulesets have the same filtering behavior, a packet matching $\mathsf {Unknown}$ in the first ruleset should also match $\lnot \;\mathsf {Unknown}$ in the second ruleset.

*Stateful Matchers in Ternary Logic.* In Sect. 4.2, we discussed the problem that some match conditions may maintain an internal state. For a match condition *a* which updates an internal state, “$a \wedge \lnot a = \mathsf {False}$” may not hold. We argued that for some state $\sigma$ and $\sigma '$, stateful match conditions need to be augmented with their internal state. For example “$a_\sigma \wedge \lnot a_{\sigma '}$”, which is not a tautology. In our implementation, we immediately embed everything in ternary logic and treat all primitives which are not definitely stateless as “unknown”. This avoids the problem with internal state and yields “$a \wedge \lnot a = \mathsf {Unknown}$”, which correctly describes the behavior since we do not know about a potential internal state of some arbitrary match condition *a*.

### Closures

In the ternary semantics, it may be unknown whether a rule applies to a packet. Therefore, the matching semantics are extended with the notion of *“in-doubt”-tactics*. A tactic is consulted if the result of a match expression is $\mathsf {Unknown}$. It decides whether a rule should apply or not.

We introduce the *in-doubt*-$allow$ and *in-doubt*-$deny$ tactics. The first tactic forces a match if the rule’s action is ${\mathtt {Accept}}$ and a mismatch if it is ${\mathtt {Drop}}$. The second tactic behaves in the opposite manner. Note that an unfolded ruleset is necessary, since no behavior can be specified for ${\mathtt {Call}}$ and ${\mathtt {Return}}$ actions.^15^

We denote the exact Boolean semantics with “$\Rightarrow$” and embedded ternary semantics with an arbitrary tactic $\alpha$ with “$\Rightarrow _\alpha$”. In particular, $\alpha$ = allow for *in-doubt*-$allow$ and $\alpha$ = deny analogously.

“$\Rightarrow$” and “$\Rightarrow _\alpha$” are related to the tactics as follows: considering the set of all accepted packets, *in-doubt*-$allow$ is an overapproximation, whereas *in-doubt*-$deny$ is an underapproximation. In other words, if “$\Rightarrow$” accepts a packet, then “$\Rightarrow _{{\text {allow}}}$” also accepts the packet. Thus, from the opposite perspective, the *in-doubt*-$allow$ tactic can be used to guarantee that a packet is certainly dropped. Likewise, if “$\Rightarrow$” denies a packet, then “$\Rightarrow _{{\text {deny}}}$” also denies this packet. Thus, the *in-doubt*-$deny$ tactic can be used to guarantee that a packet is certainly accepted.

For example, the unfolded firewall of Fig. 1 contains rules which drop a packet if a limit is exceeded. If this rate limiting is not understood by $\gamma$, the *in-doubt*-$allow$ tactic will never apply this rule, while with the *in-doubt*-$deny$ tactic, it is applied universally.

We say that the Boolean and the ternary matchers agree if they return the same result or the ternary matcher returns $\mathsf {Unknown}$. Interpreting this definition, the ternary matcher may always return $\mathsf {Unknown}$ and the Boolean matcher serves as an oracle knowing the correct result. Note that we never explicitly specify anything about the Boolean matcher; therefore the model is universally valid, i.e., the proof holds for an arbitrary oracle.

If the exact and ternary matcher agree, then the set of all packets allowed by the *in-doubt*-$deny$ tactic is a subset of the packets allowed by the exact semantics, which in turn is a subset of the packets allowed by the *in-doubt*-$allow$ tactic.^16^ Therefore, we call all packets accepted by $\Rightarrow _{{\text {deny}}}$ the *lower closure*, i.e., the semantics which accepts at most the packets that the exact semantics accepts. Likewise, we call all packets accepted by $\Rightarrow _{{\text {allow}}}$ the *upper closure*, i.e., the semantics which accepts at least the packets that the exact semantics accepts. Every packet which is not in the upper closure is guaranteed to be dropped by the firewall.

#### Theorem 5

(Lower and upper closure of allowed packets) 
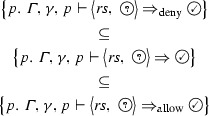


The opposite holds for the set of denied packets.^17^

For the example in Fig. 1, we computed the closures (without the RELATED,ESTABLISHED rule, see Sect. 6.4) and a ternary matcher which only understands IP addresses and layer 4 protocols. The lower closure is the empty set since rate limiting could apply to any packet. The upper closure is the set of packets originating from 192.168.0.0 / 16.

### Removing Unknown Matches

In this section, as a final optimization, we remove all unknown primitives. We call this algorithm $\mathsf {pu}$ (“process unknowns”). For this step, the specific ternary matcher and the choice of tactic must be known.

In every rule, top-level unknown primitives can be rewritten to ${\mathsf {Any}}{}$ or $\lnot \, {\mathsf {Any}}{}$. For example, let $m_u$ be a primitive which is unknown to $\gamma$. Then, for *in-doubt*-$allow$, $(m_u,\, {\mathtt {Accept}})$ is equal to $({\mathsf {Any}}{},\, {\mathtt {Accept}})$ and $(m_u,\, {\mathtt {Drop}})$ is equal to $(\lnot \,{\mathsf {Any}}{},\,{\mathtt {Drop}})$. Similarly, negated unknown primitives and conjunctions of (negated) unknown primitives can be rewritten.

Hence, the base cases of $\mathsf {pu}$ are straightforward. However, the case of a negated conjunction of match expressions requires some care. The following equation represents the De Morgan rule, specialized to the *in-doubt*-$allow$ tactic. 
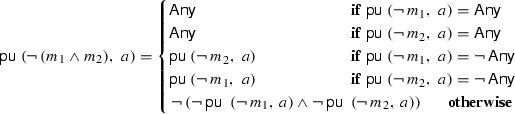


The algorithm explicitly works on ‘${\mathsf {Any}}{}$’ instead of ‘$\mathsf {True}$’, since in this context, ${\mathsf {Any}}{}$ is the syntactic base case of a match expression $M_X$ and not a Boolean or ternary value. The $\lnot \;\mathsf {Unknown} = \mathsf {Unknown}$ equation is responsible for the complicated nature of the De Morgan rule. Fortunately, we machine-verified all our algorithms.^18^ Anecdotally, we initially wrote a seemingly simple (but incorrect) version of $\mathsf {pu}$ and everybody agreed that the algorithm looks correct. In the early empirical evaluation, with yet unfinished proofs, we did not observe our bug. Only because of the failed correctness proof did we realize that we introduced an equation that only holds in Boolean logic.

#### Theorem 6

($\mathsf {pu}$ sound and complete) 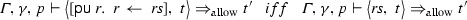


#### Theorem 7

Algorithm $\mathsf {pu}$ removes all unknown primitive match expressions.

An algorithm for the *in-doubt*-$deny$ tactic (with the same equation for the De Morgan case) can be specified in a similar way. Thus, $\Rightarrow _{\alpha }$ can be treated as if it were defined only on Boolean logic with only known match expressions.

As an example, we examine the ruleset of the upper closure of Fig. 1 (without the RELATED, ESTABLISHED rule, see Sect. 6.4) for a ternary matcher which only understands IP addresses and layer 4 protocols. The ruleset is simplified to $[({\texttt {src 192.168.0.0/16}} ,\, {\mathtt {Accept}}),\, ({\mathsf {Any}}{},\, {\mathtt {Drop}})]$. ITVal can now directly compute the correct results on this ruleset.

### The RELATED, ESTABLISHED Rule

Since firewalls process rules sequentially, the first rule has no dependency on any previous rules. Similarly, rules at the beginning have few dependencies on other rules. Therefore, firewall rules in the beginning can be inspected manually, whereas the complexity of manual inspection increases with every additional preceding rule.

It is good practice to start a firewall with an ESTABLISHED (and sometimes RELATED) rule [29]. This also happens in Fig. 1 after the rate limiting. The ESTABLISHED rule usually matches most of the packets [29],^19^ which is important for performance; however, when analyzing the filtering behavior of a firewall, it is important to consider how a connection can be brought to this state. Therefore, we remove this rule and only focus on the connection setup.

The ESTABLISHED rule essentially allows packet flows in the opposite direction of all subsequent rules [20]. Unless there are special security requirements (which is not the case in any of our analyzed scenarios), the ESTABLISHED rule can be excluded when analyzing the connection setup [20, Corollary 1].^20^ If the ESTABLISHED rule is removed and in the subsequent rules, for example, a primitive state NEW occurs, our ternary matcher returns Unknown. The closure procedures handle these cases automatically, without the need for any additional knowledge.

Our generic ruleset rewriting algorithms are not aware of connection state. Therefore, for our intermediate evaluation (Sect. 8), we removed ESTABLISHED rules by hand. In Sect. 11.2, we will describe our improvements which will enable support for conntrack state. There will no longer be any need to manually exclude rules. In short, we will fully support matches on conntrack state such as ESTABLISHED or NEW. The observation and argument of this section remains: for access control analysis, we focus on NEW packets.

## Normalization

Ruleset unfolding may result in non-atomic match expressions like $\lnot \,(a \wedge b)$. The iptables user interface only supports match expressions in *Negation Normal Form* (NNF).^21^ There, a negation may only occur before a primitive, not before compound expressions. For example, $\lnot \,({\texttt { src}} \, ip ) \,\wedge \, \mathtt {tcp}$ is a valid NNF formula, whereas $\lnot \,\left( \left( {\texttt { src }} \, ip \right) \,\wedge \, \mathtt {tcp}\right)$ is not. The reason is that iptables rules are usually specified on the command line and each primitive is an argument to the iptables command, for example $\texttt { ! --src}\, ip {\texttt { -p\, tcp }}$. We normalize match expressions to NNF, using the following observations:

De Morgan’s rule can be applied to match expressions, splitting one rule into two. For example, $\left[ \left( \lnot \;({\texttt { src}} \, ip \;\wedge \; {\texttt { tcp }}),\, {\mathtt {Accept}}\right) \right]$ and $[(\lnot \;{\texttt { src}} \, ip ,\; {\mathtt {Accept}}),\, (\lnot \; {\texttt { tcp }},\, {\mathtt {Accept}})]$ are equivalent. This introduces a “meta-logical” disjunction consisting of a sequence of consecutive rules with a shared action. For example, $[(m_1,\, a),\; (m_2,\, a)]$ is equivalent to $[(m_1 \vee m_2,\, a)]$.

For sequences of rules with the same action, a distributive law akin to common Boolean logic holds. For example, the conjunction of the two rulesets

$$\begin{aligned}&[(m_1,\, a),\; (m_2,\, a)]&\text {and}&[(m_3,\, a),\; (m_4,\, a)]&\end{aligned}$$

is equivalent to the ruleset

$$\begin{aligned}&[({m_1 \wedge m_3},\, a),\; ({m_1 \wedge m_4},\, a),\; (m_2 \wedge m_3,\, a),\; (m_2 \wedge m_4,\, a)] \text {.}&\end{aligned}$$

This can be illustrated with a situation where $a = {\mathtt {Accept}}$ and a packet needs to pass two firewalls in a row.

We can now construct a procedure which converts a rule with a complex match expression to a sequence of rules with match expressions in NNF. It is independent of the particular primitive matcher and the in-doubt tactic used. The algorithm $\mathsf {n}$ (“normalize”) of type $M_X \Rightarrow M_X\ list$ is defined as follows: 
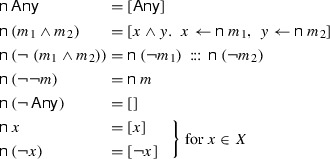
 The second equation corresponds to the distributive law, the third to the De Morgan rule. For example, $\mathsf {n}\ \left( \lnot \,({\texttt { src }}\; ip \wedge {\texttt { tcp }})\right) = \left[ \lnot \,{\texttt { src }}\; ip ,\, \lnot \, {\texttt { tcp }}\right]$. The fifth rule states that non-matching rules can be removed completely.

The unfolded ruleset of Fig. 4, which consists of nine rules, can be normalized to a ruleset of 20 rules (due to distributivity). In the worst case, normalization can cause an exponential blowup. Our evaluation shows that this is not a problem in practice, even for large rulesets. This is because rulesets are usually managed manually, which naturally limits their complexity to a level processible by state-of-the-art hardware.

### Theorem 8

$\mathsf {n}$ always terminates, all match expressions in the returned list are in NNF, and their conjunction is equivalent to the original expression.^22^

We show soundness and completeness wrt arbitrary $\gamma$, $\alpha$, and primitives.^23^ Hence, it also holds for the Boolean semantics. In general, proofs about the ternary semantics are stronger, because the ternary primitive matcher can simulate the Boolean matcher.^24^

### Theorem 9

($\mathsf {n}$ sound and complete) 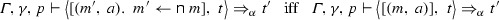


After having been normalized by $\mathsf {n}$, the rules can mostly be fed back to iptables. For some specific primitives, iptables imposes additional restrictions, e.g., that at most one primitive of a type may be present in a single rule. For our intermediate evaluation, we only need to solve this issue for IP address ranges in CIDR notation [31]. We introduced and verified another transformation which computes intersection of IP address ranges, which returns at most one range. This is sufficient to process all rulesets we encountered during our intermediate evaluation. In the following sections, we show how to support more primitives; the intermediate evaluation only focuses on IP addresses; the final evaluation (Sect. 14) incorporates many more primitives.

## Intermediate Evaluation

In this section, we demonstrate the applicability of our ruleset preprocessing described thus far. Usually, network administrators are not inclined towards publishing their firewall ruleset because of potential negative security implications. For this intermediate evaluation, we have obtained approximately $20\text {k}$ real-world rules and the permission to publish them (Sect. 17). An even larger evaluation follows in Sect. 14. In addition to the running example in Fig. 1 (a small real-world firewall), we tested our algorithms on four other real-world firewalls. We put focus on the third ruleset, because it is one of the largest and the most interesting one.

For our analysis, we wanted to know how the firewall partitions the IPv4 space. Therefore, we used a matcher $\gamma$ which only understands source/destination IP addresses and the layer 4 protocols TCP and UDP. Our algorithms do not require special processing capabilities, they can be executed within seconds on a common off-the-shelf laptop with 4GB of memory.

*Ruleset 1 * is taken from a Shorewall [28] firewall, running on a home router, with around 500 rules. We verified that our algorithms correctly unfolds, preprocesses, and simplifies this ruleset. We expected to see, in both the upper and lower closure, that the firewall drops packets from private IP ranges. However, we could not see this in the upper closure and verified that the firewall does indeed not block such packets if their connection is in a certain state. The administrator of the firewall confirmed this issue and, upon further investigation, rewrote the whole firewall.

*Ruleset 2 * is taken from a small firewall script found online [38]. Although it only contains about 50 rules, we found that it contains a serious mistake. We assume the author accidentally confused iptables’ -I (insert at top) and -A (append at tail) options. We saw this after unfolding, as the firewall allows nearly all packets at the beginning. Subsequent rules are shadowed and cannot apply. However, these rules come with a documentation of their intended purpose, such as “drop reserved addresses”, which highlights the error. We verified the erroneous behavior by installing the firewall on our systems. Thus, our unfolding algorithm alone can provide valuable insights.

*Ruleset 3 and 4 * are taken from the main firewall of our lab (Chair of Network Architectures and Services). One snapshot was taken 2013 with 2800 rules and one snapshot was taken 2014, containing around 4000 rules. It is obvious that these rulesets have grown historically. About 10 years ago, these two rulesets would have been the largest real-world rulesets ever analyzed in academia [82].

**Fig. 5:**
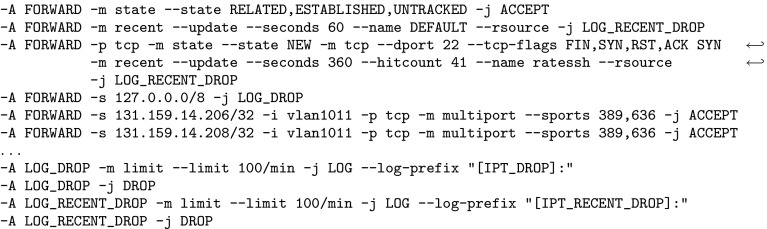
Excerpt (first rules) of the 2013 iptables ruleset of our lab

We present the analysis results of the 2013 version of the firewall. Details can be found in the additional material, the beginning of the ruleset is shown in Fig. 5. We removed the first three rules. The first rule was the ESTABLISHED rule, as discussed in Sect. 6.4. Our focus was put on the second rule when we calculated the lower closure: this rule was responsible for the lower closure being the empty set. Upon closer inspection of this rule, we realized that it was ‘dead’, i.e., it can never apply. We confirmed this observation by changing the target to a ${\mathtt {Log}}$ action on the real firewall and could never see a hit of this rule for months. Due to our analysis, this rule could be removed. The third rule performed SSH rate limiting (a ${\mathtt {Drop}}$ rule). We removed this rule because we had a very good understanding of it. Keeping it would not influence correctness of the upper closure, but lead to a smaller lower closure than necessary.

First, we tested the ruleset with the well-maintained Firewall Builder [59]. The original ruleset could not be imported by Firewall Builder due to 22 errors, caused by unknown match expressions. Using the calculated upper closure, Firewall Builder could import this ruleset without any problems.

Next, we tested ITVal’s IP space partitioning query [49]. On our original ruleset with 2800 rules, ITVal completed the query with around 3GB of RAM in around 1min. Analyzing ITVal’s debug output, we found that most of the rules were not understood correctly due to unknown primitives. Thus, the results were not reliable. We could verify this as 127.0.0.0/8, obviously dropped by our firewall, was grouped into the same class as the rest of the Internet. In contrast, using the upper and lower closure ruleset, ITVal correctly identifies 127.0.0.0/8 as its own class.

We found another interesting result about ITVal: the (optimized) upper closure ruleset only contains around 1000 rules and the lower closure only around 500 rules. Thus, we expected that ITVal could process these rulesets significantly faster. However, the opposite is the case: ITVal requires more than 10 times the resources (both CPU and RAM; we had to move the analysis to a big machine with > 40 GB of memory) to finish the analysis of the closures. We assume that this is due to the fact that ITVal now understands *all* rules. Yet, Sect. 14 will reveal that ITVal still computes wrong results.

*Limitations of the Translation.* We inspected the simplified rulesets and observed a few limitations of the translation. Those limitations mainly occur because our algorithms work on arbitrary $\gamma$. While this an important feature, it also means that we did not consider the peculiarities of specific primitives so far.

We said that iptables only accepts match expressions in NNF, but this condition alone is insufficient. In addition to NNF, each primitive must occur at most once in a match expression. For example, iptables does not allow to have two -s primitives which match on source IP addresses in an expression. However, such expressions may occur after unfolding and NNF normalization. For this intermediate evaluation, we solved this problem since we can compress the conjunction of an arbitrary number of matches on IP addresses to a single match on IP addresses: the intersection of IP address ranges in CIDR notation is either the smallest of all ranges, or the empty set (details follow in Sect. 11.1). Similarly, the conjunction of all the same matches on protocols is either the protocol itself, otherwise the match expression cannot apply to any packet and the complete rule can be removed. For example, a rule which matches on both tcp and icmp can be removed as a packet cannot be both. In addition, we see rules with ‘unknown’-parts (before the removal of unknown primitives) which can never match and should be removed. For example, it is impossible for a packet the have only the SYN and only the ACK flags set at the same time. However, without providing knowledge about tcp flags, our generic treatment of unknown match conditions may assume that this match condition may apply and such rules remain after the simplification. Hence, our simplification is still too coarse grained and loses too much information. In addition, as we indicated in Sect. 3.2, primitives may also be related and can be transformed into simpler primitives. We elaborate on the treatment of primitives in the following sections.

## Simple Firewall Model

Now, we present a very simple firewall model. This model was designed to feature nice mathematical properties, but it is too simplistic to mirror the real world. Afterwards, we will compare it to our model for real-world firewalls of Sect. 4. Section 10 will show how rulesets can be translated between these two models. This preprocessing step converts firewall rulesets from the real-world model to the simple model, which greatly simplifies all future static firewall analysis.

We will write simple firewall rules as tuple $(m,\; a)$, where *m* is a match expression and *a* is the action the firewall performs if *m* matches for a packet. The firewall has two possibilities for the filtering decision: it may accept ($\textcircled {\checkmark }$) the packet or deny () the packet. We will also use the intermediate state ($\textcircled {?}$) in which the firewall did not come to a filtering decision yet. Note that iptables firewalls always have a default policy and the $\textcircled {?}$ case cannot occur as final decision for the simple firewalls we will construct.

The semantics of the simple model is given by a recursive function. The first parameter is the ruleset the firewall iterates over, the second parameter is the packet. 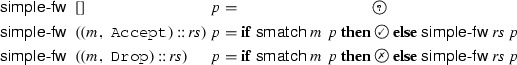


A function $\mathsf {smatch}$ tests whether a packet *p* matches the match condition *m*.^25^ The match condition is an 7-tuple, consisting of the following primitives: 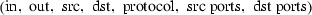 In contrast to iptables, negating matches is not supported. In detail, the following primitives are supported:

- In/out interface, including support for the ‘+’ wildcard
- Source/destination IP address range in CIDR notation, e.g., 192.168.0.0/24
- Protocol ($\mathsf {any}$, tcp, udp, icmp, or any numeric protocol identifier)
- Source/destination interval of ports, e.g., 0:65535

For example, we obtain an empty match (a match that does not apply to any packet) if and only if an end port is greater than the start port.^26^ The match which matches any packet is constructed by setting the interfaces to ‘+’, the IP to 0.0.0.0/0, the ports to 0:65535 and the protocol to $\mathsf {any}$.^27^

We require that all match conditions are well-formed, i.e., it is only allowed to match on ports (other than the universe 0:65535) if the protocol is tcp, udp, or sctp.

With this type of match expression, it is possible to implement a function $\mathsf {conj}$ which takes two match expressions $m_1$ and $m_2$ and returns exactly one match expression being the conjunction of both.^28^

### Theorem 10

(Conjunction of two simple match expressions) 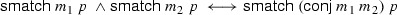


Computing the conjunction of the individual match expressions for port intervals and single protocols is straightforward. The conjunction of two intervals in CIDR notation is either empty or the smaller of both intervals. The conjunction of two interfaces is either empty if they do not share a common prefix, otherwise it is the longest of both interfaces (non-wildcard interfaces dominate wildcard interfaces).

The $\mathsf {conj}$ of two well-formed matches is again well-formed.^29^

The type of match expressions was carefully designed such that the conjunction of *two* match expressions is only *one* match expression. If features were added to the match expression, for example negated interfaces, this property would no longer be guaranteed. Of the features most commonly found in our iptables firewall rulesets [3], we only found that it would further be possible to add TCP flags to the match expression without violating the aforementioned conjunction property. Considering common features of firewalls in general [70], it would probably be possible to enhance the ICMP support of our model.

One advantage of  over the semantics of Fig. 2 is that it is a simple recursive function. In addition,  is total, i.e., it is guaranteed to terminate. This is not the case for the semantics of Fig. 2, as the assumptions of Theorem 3 show. Hence, the simple firewall makes proofs about the filtering behavior much easier as they can often be done by a list induction over the ruleset. Another advantage is that the  function of  is completely defined and it is no longer required to reason about an arbitrary but fixed function $\gamma$.

## Translation to the Simple Firewall Model

The semantics given in Sect. 4 includes a primitive matcher $\gamma$ that decides whether a certain primitive matches a packet. The model and all algorithms on top of it are proven correct for an arbitrary $\gamma$, hence, this model supports *all* iptables matching features. Obviously, there is no executable code for an arbitrary $\gamma$. However, the algorithms to transform rulesets we present are executable. To have a clear semantics of the primitives, we have defined a subset of $\gamma$, namely for all primitives supported by the simple firewall and some further primitives, detailed in Sect. 11. We assume that $\gamma$ behaves as expected on our subset, but it may show arbitrary behavior for all other primitives. We say we *agree* on $\gamma$. For example, $\gamma$ behaves as expected on IP addresses, but it may show arbitrary behavior for a bfp match.

Using our previously described algorithms, we assume that the ruleset is already unfolded and the match expressions are normalized. This leaves a ruleset where only the following actions occur: ${\mathtt {Accept}}$ and ${\mathtt {Drop}}$.^30^ Thus, a large step for translating the real-world model to the simple firewall model is already accomplished. Translating the match expressions for the simple firewall remains. Of course, it is not possible to translate all primitives to the very limited  model, so we will make use of the $\mathsf {pu}$ algorithm when necessary. For the sake of example, we will only consider the overapproximation in the following parts of this article; the underapproximation is analogous and can be found in our formalization.

Since firewalls usually accept all packets which belong to an ESTABLISHED connection, the interesting access control rules in a ruleset only apply to NEW packets. We only consider NEW packets, i.e., ${{-}{-}{} \texttt {ctstate NEW}}$ and ${{-}{-}{} \texttt {syn}}$ for TCP packets. Our first goal is to translate a ruleset from the real-world model to the simple model. We have proven that the set of new packets accepted by the simple firewall is a superset (overapproximation) of the packets accepted by the real-world model.^31^ This is a core contribution and we expand on the translation in the following section.

### Theorem 11

(Translation to simple firewall model) For $\gamma$ we *agree* on 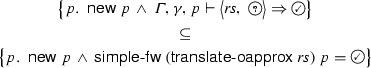


Any packet dropped by the translated, overapproximated simple firewall ruleset is guaranteed to be dropped by the real-world firewall, for arbitrary $\gamma$, $\varGamma$, $rs$. Similar guarantees for definitely accepted packets can be given by considering the translated underapproximation. Given the simplicity of the  model, it is much easier to write algorithms to analyze and verify the translated rulesets.

*Example.* Because this article proceeds to focus more on individual primitives, we will increasingly use the more precise syntax of iptables-save which is also described by the man pages iptables(8) and iptables-extensions(8). We consider a FORWARD chain with a default policy of $\mathtt {Drop}$ and a user-defined chain foo.
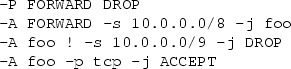


This ruleset, although it only consists of three rules and a default policy, is complicated to analyze. Our translation algorithm translates it to the simple firewall model, where the ruleset becomes remarkably simple. We use $*$ to denote a wildcard: 
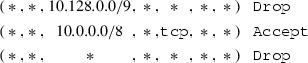
 No over- or underapproximation occurred since all primitives could be translated. Note the 10.128.0.0/9 address range, which is the result of the intersection of 10.0.0.0/8 and the negation of 10.0.0.0/9.

## Translating Primitives

In this section, we present algorithms to transform specific primitives without changing the behavior of the firewall.^32^ As a result, the primitive matches on interfaces, IP addresses, protocols, and ports will be normalized such that the translation to the  is obvious. Since iptables supports over 200 individual options for match conditions, we cannot cover all. For example, we do not support any IPsec ah or esp matches or bpf matches, but we will simply abstract over them using our algorithm $\mathsf {pu}$. However, we support the most common features found in common iptables rulesets. Simple matches, such as -s or -d to match on source or destination IP addresses are already supported by the . The iprange or multiport module allow matching on IP addresses and ports, but are more expressible than the  supports. We translate them without the loss of information, but at the cost of increased ruleset size. Other modules, such as conntrack state or tcp flags cannot be expressed in the  at all. However, we are sometimes able to rewrite them directly to ${\mathsf {Any}}$ or $\lnot \;{\mathsf {Any}}$. We continue by describing the normalization of all common primitives found in iptables rulesets.

### IPv4 Addresses

According to Nelson [54], “[m]odeling IP addresses efficiently is challenging.” First, we present a datatype to efficiently perform set operations on intervals of machine words, e.g., 32bit integers. We will use this type for IPv4 addresses, but we have generalized to machine words of arbitrary length, e.g., IPv6 addresses or layer 4 ports. For brevity, we will present our formalization at the example of IPv4. We call our datatype a word interval ($wi$), and $\mathsf {WI}\ start \ end$ describes the (inclusive) interval. The $\mathsf {Union}$ of two $wi$s is defined recursively. 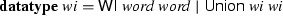 Let $\mathsf {set}$ denote the interpretation into mathematical sets, then $wi$ has the following semantics: 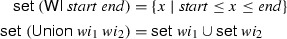


An IP address in CIDR notation or IP addresses specified by e.g., ${-\texttt {m iprange}}$ can be translated to a single $\mathsf {WI}$ value. We have implemented and proven correct the common set operations: ‘$\cup$’, ‘$\lbrace \rbrace$’, ‘$\setminus$’, ‘$\cap$’, ‘$\subseteq$’, and ‘$=$’. These operations are linear in the number of $\mathsf {Union}$ constructors. The result is optimized by merging adjacent and overlapping intervals and removing empty intervals. We can also represent ‘$\mathsf {UNIV}$’ (the universe of all IP addresses). Since most rulesets use IP addresses in CIDR notation or intervals in general, the $wi$ datatype has proven to be very efficient. Recall that the intersection of two intervals, constructed from addresses in CIDR notation, is either empty or the smaller of both intervals.^33^

The datatype $wi$ is an internal representation and for the simple firewall, the result needs to be represented in CIDR notation. For this direction, one $\mathsf {WI}$ may correspond to several CIDR ranges. We describe an algorithm to $\mathsf {split}$ off one CIDR range from an arbitrary word interval *r*. The output is a CIDR range and $r^\prime$, the remainder after splitting off this CIDR range. $\mathsf {split}$ is implemented as follows: let $a$ be the lowest element in *r*. If this does not exist, then *r* corresponds to the empty set and the algorithm terminates. Otherwise, we construct the list of CIDR ranges [*a* / 0, *a* / 1, ..., *a* / 32]. The first element in the list which is well-formed (i.e., all bits after the network prefix must be zero) and which is a subset of *r* is the wanted element. Note that this element always exists. It is subtracted from *r* to obtain $r^\prime$. To convert *r* completely to a list of CIDR ranges, this is applied recursively until it yields no more results. This algorithm is guaranteed to terminate and the resulting list in CIDR notation corresponds to the same set of IP addresses as represented by *r*.^34^ Formally, $\bigcup \mathsf {map}\ \mathsf {set}\ (\mathsf {split}\ r) = \mathsf {set}\ r$.

*Example.* 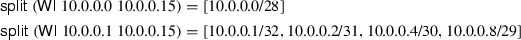


With the help of these functions, arbitrary IP address ranges can be translated to the format required by the simple firewall. The following is applied to matches on source and destination IP addresses: first, the IP match expression is translated to a word interval. If the match on an IP range is negated, we compute $\mathsf {UNIV} \setminus wi$. All matches in one rule can be joined to a single word interval, using the $\cap$ operation. The resulting word interval is translated to a set of non-negated CIDR ranges. Using the NNF normalization, at most one match on an IP range in CIDR notation remains. We have proven that this process preserves the firewall’s filtering behavior.

We conclude with a simple, artificial worst-case example. The evaluation shows that it does not prevent successful analysis: $-\texttt {m iprange} {-}{-}{} \texttt {src-range 0.0.0.1-255.255.}{} \texttt {255.254}$. Translated to the simple firewall, this one range blows up to 62 ranges in CIDR notation. A similar blowup may occur for negated IP ranges.

Note that, while pretty printing IPv4 addresses in dotecimal notation (i.e., $\texttt {<dotnum>}{} \texttt { :{:}=<snum> }{{\prime {\prime }}}.{{\prime {\prime }}}{} \texttt {<snum> }{\prime {\prime }}.{\prime {\prime }} \texttt {<snum> } {\prime {\prime }}.{\prime {\prime }} \texttt { <snum> }$ [75]) is simple, pretty printing IPv6 addresses is non-trivial [40] and our implementation contains the first formally machine-verified IPv6 pretty printer [24].

### conntrack State

If a packet *p* is matched against the stateful match condition ESTABLISHED, conntrack looks up *p* in its state table. When the firewall comes to a filtering decision for *p*, if the packet is not dropped and the state was NEW, the conntrack state table is updated such that the flow of *p* is now ESTALISHED. Similarly, other conntrack states are handled.

We present an alternative model for this behavior: before the firewall starts processing the ruleset for *p*, the conntrack state table is consulted for the state of the connection of *p*. This state is added as a (phantom) tag to *p*. Therefore, ctstate can be modeled as just another header field of *p*. When processing the ruleset, it is not necessary to inspect the conntrack table but only the virtual state tag of the packet. After processing, the state table is updated accordingly.

We have proven that both models are equivalent to each other.^35^ The latter model is simpler for analysis purposes since the conntrack state can be considered an ordinary packet field.^36^

In Theorem 11, we are only interested in NEW packets. In contrast to our intermediate evaluation (Sect. 8), there is no longer the need to manually exclude ESTABLISHED rules from a ruleset. The alternative model allows us to consider only NEW packets: all state matches can be removed (by being pre-evaluated for an arbitrary NEW packet) from the ruleset without changing the filtering behavior of the firewall.

### Layer 4 Ports

Translating singleton ports or intervals of ports to the simple firewall is straightforward. A challenge remains for negated port ranges and the multiport module. However, the word interval type is also applicable to 16bit machine words and solves these challenges. For ports, there is no need to translate an interval back to CIDR notation.^37^

In the original paper [23], we made a serious mistake [27] when specifying the semantics of matches on ports. Fortunately, the error only manifests itself in corner cases and did not affect the published evaluation. However, we have seen rulesets in the wild which triggered the bug, hence, it is not purely of academic nature. Since we have proven the correctness of all our algorithms and checked all assumptions, the bug did not exist in the code, but in the model. We describe the problem and its resolution (which has already been implemented) in this section.

We defined the datatype of a source port match as follows: 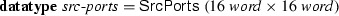


This datatype describes a source port match as an interval of 16 bit port numbers. The match semantics for a packet were defined such that the source port of the packet must be in the interval. For example, packet *p* matches $\mathsf {SrcPorts}\ a\ b$ if and only if 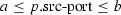. We defined $\mathsf {DstPorts}$ analogously. With these semantics, we can construct a corner case which describes why this model does not correspond to reality. Consider the following firewall:
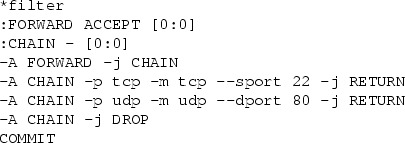


The firewall in iptables-save format shows the filter table, which consists of the two chains FORWARD and CHAIN. The FORWARD chain is built-in and has a default policy of Accept here. Starting at the FORWARD chain, any packet which is processed by this firewall is directly sent to the user-defined chain CHAIN first. A packet can only Return if it is a TCP packet with source port 22 or a UDP packet with destination port 80. All other packets are dropped. Hence, this firewall expresses in a complicated way the following policy: *“Drop everything which is not* tcp *source port 22 or* udp *destination port 80”*. This ruleset, though it does not have an obvious use, was artificially constructed to demonstrate our bug. Our tool has “simplified” the ruleset in the following way:
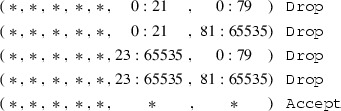
 Given our semantics, the simplification is correct. In reality, this simple firewall is wrong for various reasons. First, it is not well-formed, i.e., it tries to match on ports without specifying a protocol. Second, it has mixed up UDP and TCP ports.

The problem lies in our semantics of $\mathsf {SrcPorts}$ and $\mathsf {DstPorts}$. Roughly speaking, there is no such a thing as “ports”, but TCP ports, UDP ports, SCTP ports, and many others.

We have resolved the issue by including the protocol in the match for a port: 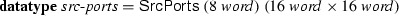


The $8\ word$ corresponds to the protocol field in IPv4 [68], respectively the Next Header field in IPv6 [17], identifying protocols by their assigned numbers [72, 73]. It does not allow a wildcard. The semantics defines that the protocol of a packet must be the same as specified in the datatype and that the source port must be in the interval (as in the first definition).

With the corrected semantics, our tool computes the correct and expected result: 
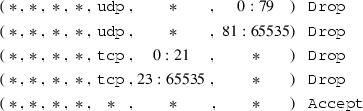
 The negation of a match on ports is the interesting corner case to which the presented problems can be reduced to. We will illustrate the issue with a simpler example. Assuming we have one rule which tries to accept every packet which is not udp destination port 80.^38^ For simplicity, we assume we have one rule as follows: ! (-p udp --dport 80) -j ACCEPT . Semantically, to unfold this negation, the rule matches either everything which is not udp or everything which is udp but not destination port 80. It can be expressed with the following two rules: ! -p udp -j ACCEPT followed by -p udp ! --dport 80 -j ACCEPT . We use this strategy in our tool to unfold the negation of matches on ports. Note the type dependencies which occur: negating one rule that matches on ports yields both a rule which matches on protocols and one rule which matches on ports.

This example also shows that any tool which reduces match conditions to a flat bit vector is either flawed (it loses the protocol which belongs to a match on ports) or cannot support complicated negations. This includes tools which reduce firewall analysis to SAT [39] or BDDs [1, 85]. It may probably also affect ITVal [48] which relies on multi-way decision diagrams (MDD). This was also the case for our ${\varGamma ,\gamma ,p \vdash \big \langle rs ,\; s \big \rangle \Rightarrow t}$ semantics with the flawed $\gamma$ described here. Our simple firewall model does not allow complicated negations and we have proven that the match conditions are always well-formed, hence, the presented class of errors cannot occur there.

### TCP Flags

iptables can match on a set of layer 4 flags. To match on flags, a $mask$ selects the corresponding flags and *c* declares the flags which must be present. For example, the match --syn is a synonym for $mask =\mathtt {SYN,RST,ACK,FIN}$ and $c=\mathtt {SYN}$. For a set *f* of flags in a packet, matching can be formalized as $f \cap mask = c$. If *c* is not a subset of $mask$, the expression cannot match; we call this the *empty match*. We proved that two matches $( mask _1, c_1)$ and $( mask _2, c_2)$ are equal if and only if $( \mathbf {if}\ c_1 \subseteq mask _1 \wedge c_2 \subseteq mask _2 \ \mathbf {then}\ c_1 = c_2 \wedge mask _1 = mask _2 \ \mathbf {else}\ (\lnot c_1 \subseteq mask _1) \wedge (\lnot c_2 \subseteq mask _2))$ holds. We also proved that the conjunction of two matches is exactly $( \mathbf {if}\ c_1 \subseteq mask _1 \wedge c_2 \subseteq mask _2 \wedge mask _1 \cap mask _2 \cap c_1 = mask _1 \cap mask _2 \cap c_2\ \mathbf {then}\ ( mask _1 \cup mask _2,\, c_1 \cup c_2) \ \mathbf {else}\ \mathtt {empty})$. If we assume --syn for a packet, we can remove all matches which are equal to --syn and add the --syn match as conjunction to all other matches on flags and remove rules with empty matches. Some matches on flags may remain, e.g., $\mathtt {URG}$, which need to be abstracted over later.

### Interfaces

The simple firewall model does not support negated interfaces, e.g., ! -i eth+. Therefore, they must be removed. We first motivate the need for abstracting over negated interfaces.

For whitelisting scenarios one might argue that the use of negated interfaces constitutes bad practice. This is because new (virtual) interfaces might be added to the system at runtime and a match on negated interfaces might now also include these new interfaces. Therefore, negated interfaces correspond to blacklisting, which is not recommended for most firewalls. However, the main reason why negated interfaces are not supported by our model is of technical nature: let $\mathsf {set}$ denote the set of interfaces that match an interface expression. For example, $\mathsf {set}\ {\texttt {eth0}} = \lbrace {\texttt {eth0}}\rbrace$ and $\mathsf {set}\ {\texttt {eth+}}$ is the set of all interfaces that start with the prefix eth. If the match on ${\texttt {eth+}}$ is negated, then it matches all strings in the complement set: $\mathsf {UNIV} \setminus (\mathsf {set}\ {\texttt {eth+}})$. The simple firewall model requires that a conjunction of two primitives is again at most one primitive. This can obviously not be achieved with such sets. In addition, working with negated interfaces can cause great confusion. Note that the interface match condition ‘+’ matches any interfaces. Also note that ‘+’ $\in \mathsf {UNIV} \setminus (\mathsf {set}\ {\texttt {eth+}})$. Here, ‘+’ is not a wildcard character but the name of an interface. The confusion introduced by negated interfaces becomes more apparent when one realizes that ‘+’ can occur as both wildcard character and normal character. Therefore, it is not possible to construct an interface match condition which matches exactly on the interface ‘+’, because a ‘+’ at the end of an interface match condition is interpreted as wildcard.^39^ While technically, the Linux kernel would allow to match on ‘+’ as a normal character [46], the iptables command does not permit to construct such a match [60].

### Interaction of Interfaces with IP Ranges

Later, in Sect. 12.1, we will compute an IP address space partition. For better understanding, that partition should not be “polluted” with interface information. Therefore, for the partition, we will assume that no matches on interfaces occur in the ruleset. In this section, we describe a method to remove both negated and non-negated interfaces while preserving their relation to IP address ranges.

Input interfaces are usually assigned an IP range of valid source IPs which are expected to arrive on that interface. Let $$ ipassmt $$ be a mapping from interfaces to an IP address range. This information can be obtained by ip route and ip addr. We will write $$ ipassmt [i]$$ for the corresponding IP range of interface *i*. For the following examples, we assume 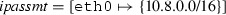 The goal is to rewrite input interfaces with the corresponding source IP range. For example, we would like to replace all occurrences of -i eth0 with -s 10.8.0.0/16. This idea can only be sound if there are no spoofed packets; we only expect packets with a source IP of 10.8.0.0/16 to arrive at eth0. Once we have assured that the firewall blocks spoofed packets, we can assume in a second step that there are no spoofed accepted packets left. By default, the Linux kernel offers reverse path filtering, which blocks spoofed packets automatically. In this case we can assume that no spoofed packets occur. In some complex scenarios, reverse path filtering needs to be disabled and spoofed packets should be blocked manually with the help of the firewall ruleset. In previous work [26], we presented an algorithm to verify that a ruleset correctly blocks spoofed packets. This algorithm is integrated in our framework, proven sound, works on the same $ipassmt$, and does not need the simple firewall model (i.e., supports negated interfaces). If some interface *i* should accept arbitrary IP addresses (essentially not providing spoofing protection), it is possible to set $ipassmt [i] = \mathsf {UNIV}$. Therefore, we can verify spoofing protection according to $ipassmt$ at runtime and afterwards continue with the assumption that no spoofed packets occur.

Under the assumption that no spoofed packets occur, we will now present two algorithms to relate an input interface *i* to $$ ipassmt [i]$$. Both approaches are valid for negated and non-negated interfaces. The first approach provides better results but requires stronger assumptions (which can be checked at runtime), whereas the second approach can be applied without further assumptions.

*First Approach.* In general, it is considered bad practice [82, 83] to have zone-spanning interfaces. Two interfaces are zone-spanning if they share a common, overlapping IP address range. Mathematically, absence of zone-spanning interfaces means that for any two interfaces in $ipassmt$, their assigned IP range must be disjoint. Our tool emits a warning if $ipassmt$ contains zone-spanning interfaces. If no zone-spanning interfaces are detected, then all input interfaces can be replaced by their assigned source IP address range. This preserves exactly the behavior of the firewall. In this case, there is an injective mapping between input interfaces and source IPs. Interestingly, our proof does not need the assumption that $ipassmt$ maps to the complete IP universe.

*Second Approach.* Unfortunately, though considered bad practice, we found zone-spanning interfaces in many real-world configurations and hence cannot apply the previous algorithm. First, we proved that correctness of the described rewriting algorithm implies absence of zone-spanning interfaces.^40^ This leads to the conclusion that it is impossible to perform rewriting without this assumption. Therefore, we present an algorithm which adds the IP range information to the ruleset (without removing the interface match), thus constraining the match on input interfaces to their IP range. The algorithm computes the following: whenever there is a match on an input interface *i*, the algorithm looks up the corresponding IP range of that interface and adds -s $$ ipassmt [i]$$ to the rule. To prove correctness of this algorithm, no assumption about zone-spanning interfaces is needed, $$ ipassmt $$ may only be defined for a subset of the interfaces, and the range of $$ ipassmt $$ may not cover the complete IP universe. Consequently, there is no need for a user to specify $$ ipassmt $$, but having it may yield more accurate results.

*Output Port Rewriting.* Our presented approaches for input interface rewriting can be generalized to also support output interface (-o) rewriting. The core idea is to replace a match on an output interface by the corresponding IP address range which is determined by the system’s routing table. To do this, we parse the routing table, map it to a relation (which provides a structure which is independent of its order), and compute the inverse of the relation. This ultimately provides a mapping for each interface and its corresponding IP address range.

This computed mapping is very similar to the $ipassmt$. In fact, we found it to be a helpful debugging tool to compare the inverse routing relation to an $ipassmt$. For convenience, we also provide a function to compute an $ipassmt$ from a routing table.

Essentially, computing the inverse routing relation semantically is the same behavior as found in strict reverse path filtering [5]. We have formally proven^41^ this observation.

**Table 1:** Summary of evaluation on real-world firewalls

Fw	Rules	Chain (unfolded)	Simple rules (no ifaces)	Use	Parts (ITVal)	SSH	HTTP	Time (ITVal)	Time (this) [s]
A	2784	FW (2376)	2381 (1920)		246 (1)	13	9	3 $\hbox {h}^\mathrm{a}$	172
	–	FW (2376)	2837 (581)		522 (1)	1	1	9 $\hbox {h}^\mathrm{a}$	194
A	4113	FW (2922)	3114 (2862)		334 (2)	11	11	27 $\hbox {h}^\mathrm{a}$	302
	–	FW (2922)	3585 (517)		490 (1)	1	1	8 h	320
A	4814	FW (4403)	3574 (3144)		364 (2)	9	12	46 $\hbox {h}^\mathrm{a}$	477
	–	FW (4403)	5123 (1601)		1574 (1)	1	1	3 $\hbox {h}^\mathrm{a}$	618
A	4946	FW (4887)	4004 (3570)		371 (2)	9	12	53 $\hbox {h}^\mathrm{a}$	578
	–	FW (4887)	5563 (1613)		1585 (1)	1	1	4 $\hbox {h}^\mathrm{a}$	820
B	88	FW (40)	110 (106)		50 (4)	4	2	2 s	3
	–	FW (40)	183 (75)		40 (1)	1	1	1 s	2
C	53	FW (30)	29 (12)		8 (1)	1	1	1 s	1
	–	FW (30)	27 (1)		1 (1)	1	1	1 s	1
	–	IN (49)	74 (46)		38 (1)	1	1	1 s	1
	–	IN (49)	75 (21)		6 (1)	1	1	1 s	1
D	373	FW (2649)	3482 (166)		43 (1)	1	1	3 s	22
	–	FW (2649)	16592 (1918)		67 (1)	1	1	33 $\hbox {min}^\mathrm{a}$	49
E	31	IN (24)	57 (27)		4 (3)	1	2	1 s	10
	–	IN (24)	61 (45)		3 (1)	1	1	1 s	1
F	263	IN (261)	263 (263)		250 (3)	3	3	2 min	80
	–	IN (261)	265 (264)		250 (3)	3	3	3 min	57
G	68	IN (28)	20 (20)		8 (5)	1	2	1 s	8
	–	IN (28)	19 (19)		8 (2)	2	2	1 s	1
H	19	FW (20)	10 (10)		9 (1)	1	1	1 s	8
	–	FW (20)	8 (8)		3 (1)	1	1	1 s	1
I	15	FW (5)	4 (4)		4 (4)	4	4	1 s	8
	–	FW (5)	4 (4)		4 (4)	4	4	1 s	1
J	48	FW (12)	5 (5)		3 (2)	2	2	1 s	6
	–	FW (12)	8 (2)		1 (1)	1	1	1 s	1
K	21	FW (9)	7 (6)		3 (1)	1	1	1 s	12
	–	FW (9)	4 (3)		2 (1)	1	1	1 s	1
L	27	IN (16)	19 (19)		17 (3)	2	2	1 s	1
	–	IN (16)	18 (18)		17 (3)	2	2	1 s	1
M	80	IN (92)	64 (16)		2 (2)	1	2	1 s	6
	–	IN (92)	58 (27)		11 (1)	1	1	1 s	1
N	34	FW (14)	12 (12)		10 (6)	6	6	2 s	2
	–	FW (14)	12 (12)		10 (6)	6	6	2 s	1
O	8	IN (7)	9 (9)		3 (3)	1	2	1 s	1
	–	IN (7)	8 (8)		3 (3)	1	2	1 s	1
P	595	IN (15)	8 (8)		3 (2)	2	2	1 s	6
	–	IN (15)	9 (9)		3 (2)	2	2	1 s	6
	595	FW (66)	64 (64)		60 (5)	5	4	22 s	6
	–	FW (66)	63 (63)		60 (5)	5	4	22 s	7
Q	58	IN (59)	65 (65)		21 (1)	1	1	2 s	2
	–	IN (59)	62 (62)		21 (2)	2	1	2 s	1
R	30	FW (28)	123 (123)		14 (1)	1	6	1 s	1
	–	FW (28)	20 (3)		2 (2)	2	1	1 s	1

${}^\mathrm{a}$ ITVal memory consumption, in order of appearance: 84, 96, 94, 95, 61, 98, 96 and 21 GB

Because a routing table may change frequently, even triggered by external malicious routing advertisements, by default, we refrain from output port rewriting in this work. In general, we will not apply it in our evaluation (Sect. 14, Table 1); however, in one case (Sect. 14, Firewall D) we will additionally show how the results improve.

### Abstracting Over Primitives

Some primitives cannot be translated to the simple model. Section 6.3 already provides the function $\mathsf {pu}$ which removes all unknown match conditions. This leads to an approximation and is the main reason for the ‘$\subseteq$’ relation in Theorem 11. We found that we can also rewrite any known primitive at any time to an unknown primitive. This can be used to apply additional knowledge during preprocessing. For example, since we understand flags, we know that the following condition is false, hence rules using it can be removed: --syn $\wedge$ --tcp-flags RST,ACK RST. After this optimization, all remaining flags can be treated as unknowns and abstracted over afterwards. This allows to easily add additional knowledge and optimization strategies for further primitive match conditions without the need to adapt any algorithm which works on the simple firewall model. We proved soundness of this approach: the ‘$\subseteq$’ relation in Theorem 11 is preserved.

## Analyzing Simple Firewall Rulesets

In this section, we will show two algorithms that work on rulesets translated to the  model.

### IP Address Space Partitioning

We present an algorithm to partition the full space of IP addresses into equivalence classes. It runs roughly in linear time in the number of rules for real-world rulesets. All IP addresses in the same partition show the same behavior wrt the firewall ruleset. We do not require that the partition is minimal. Therefore, the following would be a valid solution: $\left\{ \left\{ 0 \right\} ,\ \left\{ 1 \right\} ,\ \dots ,\ \left\{ 255.255.255.255 \right\} \right\}$. However, we will need the partition as starting point for a further algorithm and a partition of size ${2}^{32}$ (in case of IPv4) is too large for this purpose. In the case of IPv6, one address per partition would be infeasible.

First, we motivate the idea of the partitioning algorithm with the following observation. For an arbitrary packet *p*, we write $p(\mathtt {src} \mapsto s)$ to fix the source IP address to *s*.

#### Lemma 1

Let *X* be the set of all source IP matches specified in $rs$, i.e., *X* is a set of CIDR ranges. Given that we have a set *B* such that $\forall A \in X.\ B \subseteq A \vee B \cap A = \lbrace \rbrace$ holds. Then, for $s_1 \in B$ and $s_2 \in B$, 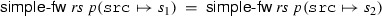


Reading the lemma backwards, it states that all packets with arbitrary source IPs picked from *B* are treated equally by the firewall. Therefore, *B* is a member of an IP address range partition. The condition imposed on *B* is that for all source CIDR ranges which are matched on in the ruleset (called *A* in the lemma), *B* is either a subset of the range or disjoint to it. The lemma shows that this condition is sufficient for *B*, therefore we will construct an algorithm to compute *B*. For an arbitrary set *X*, this condition is purely set-theoretic and we can solve it independently from the firewall theory. For simplicity, we use finite sets and lists interchangeably.

The algorithm $\mathsf {partitions}$ is structured as follows. The $\mathsf {part}$ function computes a single step and takes two parameters. The first parameter is a set $S \in X$, the second parameter $TS$ is a set of sets and corresponds to the remaining set which will be partitioned. In the first call, we set $TS$ to $\lbrace \mathsf {UNIV} \rbrace$. Then, we repeatedly call $\mathsf {part}$ on all elements in *X* and thread through the results, i.e., 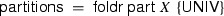


The step function $\mathsf {part}$ itself is implemented as follows: for a fixed *S*, $\mathsf {part} \ S \ TS$ recurses over $TS$ and splits the set such that the precondition of Lemma 1 holds. 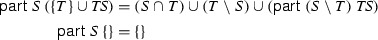


The result size of calling $\mathsf {part}$ once can be up to two times the size of $TS$. This implies that the size of the partition of a complete firewall ruleset is in the order of $O(2^{\vert rules \vert })$. However, the empirical evaluation shows that the resulting size for real-world rulesets is much better. While IP address ranges may overlap in a ruleset, they usually do not overlap in the worst possible way for all pairs of rules. Consequently, at least one of the sets $S \cap T$ or $T \setminus S$ is usually empty. For example, for our largest firewall, the number of computed partitions is 10 times smaller than the number of rules. Our evaluation (Table 1 in Sect. 14) confirms that the number of partitions is usually less than the number of rules.

Our algorithm fulfills the assumption of Lemma 1 for arbitrary *X*. Because IP addresses occur as source and destination in a ruleset, we use our partitioning algorithm where *X* is the set of all IPs found in the ruleset. The result is a partition where for any two IPs in the same partition, setting the source or destination of an arbitrary packet to one of the two IPs, the firewall behaves equally. This results in a stronger version of Lemma 1, which holds without any assumption and also holds for both source and destination IPs simultaneously.^42^ In addition, the partition covers the complete IPv4 (or IPv6) address space.^43^

### Service Matrices

The computed IP address space partition may not be minimal. This means that two different partitions may exhibit exactly the same behavior. Therefore, for manual firewall verification, these partitionings may be misleading. Marmorstein elaborates on this problem [49]. ITVal’s solution is to minimize the partitioning. We suggest to minimize the partitioning, but wrt a fixed service. The evaluation shows that the result is smaller and thus clearer.

**Fig. 6:**
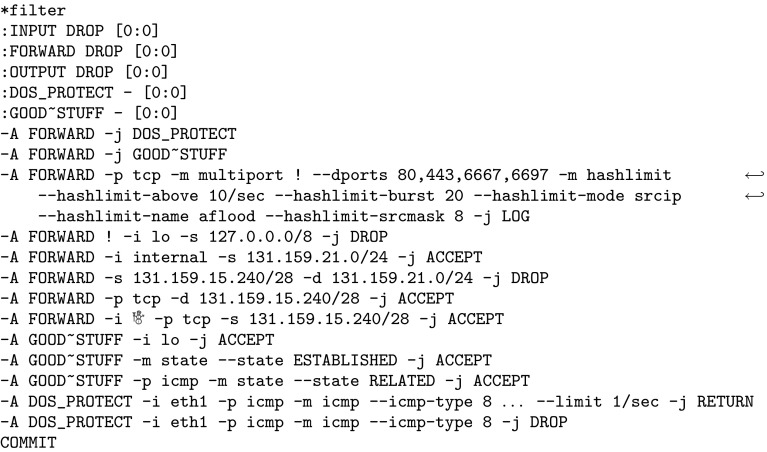
Example ruleset. (Color figure online)

**Fig. 7:**
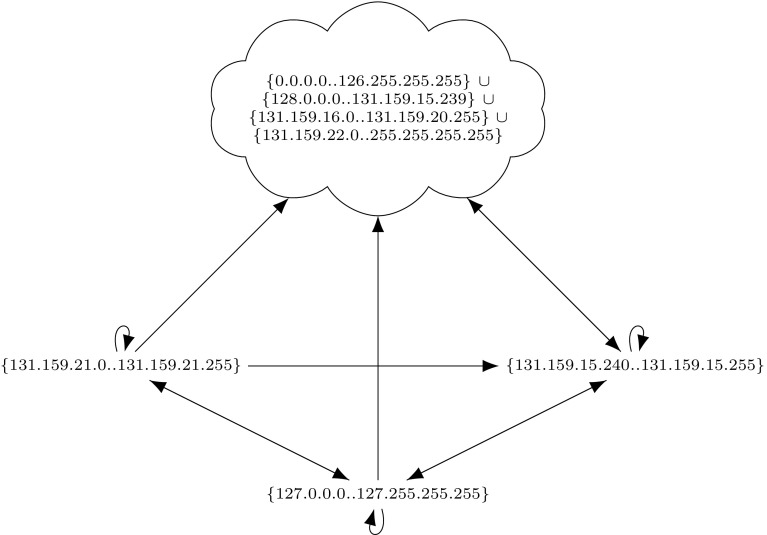
Service matrix of ruleset in Fig. 6

**Fig. 8:**
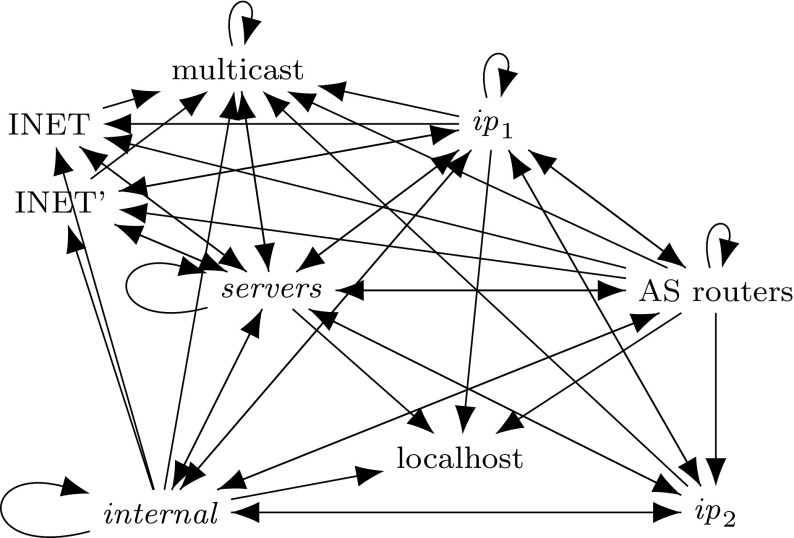
Lab SSH Service Matrix (2015)

**Fig. 9:**
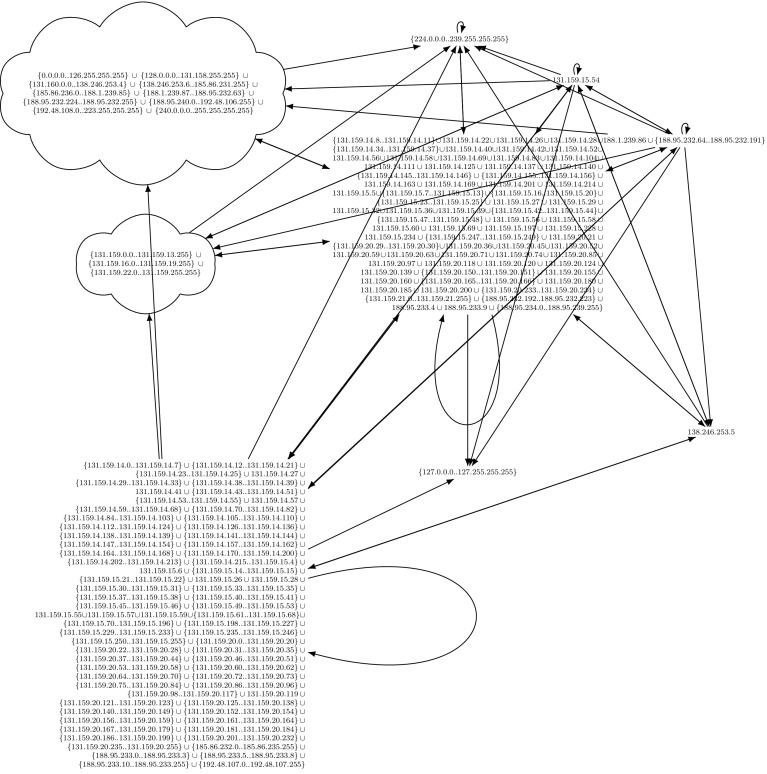
Lab SSH Service Matrix with raw IP addresses (2015)

**Fig. 10:**
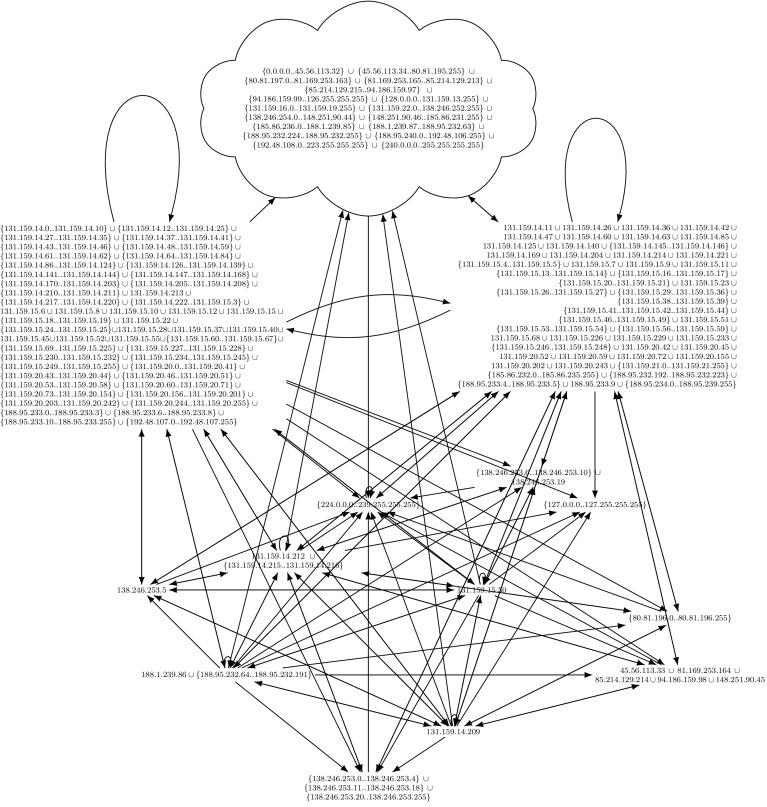
Lab IPv4 HTTP Service Matrix (2016)

**Fig. 11:**
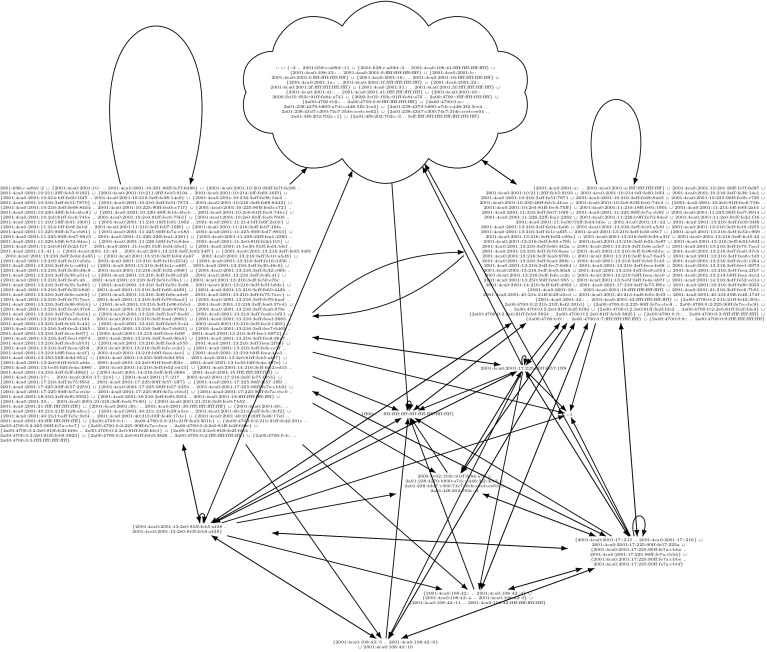
Lab IPv6 HTTP Service Matrix (2016)

A fixed service corresponds to a fixed packet with arbitrary IPs. For example, we can define SSH as TCP, destination port 22, and arbitrary but fixed source port $\ge$ 1024. A service matrix describes the allowed accesses for a specific service over the complete IPv4 (or IPv6) address space. It can be visualized as a graph; for example, the ruleset of Fig. 6 is visualized in Fig. 7. An example of a service matrix for a firewall with several thousands of rules is shown in Fig. 8. For clarity, this figure uses symbolic names (e.g., $servers$) instead of IP addresses. The raw IP addresses can be found in Fig. 9. More complicated examples with highly fragmented IP ranges are shown in Figs. 10 and 11; those stem from the same firewall installation, but at a later time. All matrices are minimal, i.e., they cannot be compressed any further.

First, we describe when a firewall exhibits the same behavior for arbitrary source IPs $s_1, s_2$ and a fixed packet *p*: 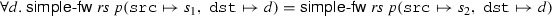 We say the firewall shows same behavior for a fixed service if, in addition, the analogue condition holds for destination IPs.

We present a function $\mathsf {groupWIs}$, which computes the minimal partitioning for a fixed service. The idea is to start with the output of the algorithm $\mathsf {partitions}$ and minimize it. For this, the full, square access control matrix for inbound and outbound connections of each partition member is generated. An entry $m_{i,j}$ in this matrix denotes whether partition member *i* is allowed to communicate with partition member *j*. In detail, an entry $m_{i,j}$ is a pair of Boolean values, where the first element denotes whether all IP addresses in *i* are allowed to communicate with all IP addresses in *j* and the second entry denotes whether all IP addresses in *j* are allowed to communicate with all IP addresses in *i*. To compute all the entries $m_{i,j}$, the algorithm performs two calls (one for source IP and one for destination IP) to  for each pair of partition members. This can be done by taking arbitrary representatives from each member of the partition as source and destination address and executing  for the fixed packet with those fixed IPs. The matrix is minimized by merging partitions with equal behavior, i.e., merging equal rows in the matrix. This algorithm is quadratic in the number of partitions. An early evaluation [23] shows that it scales surprisingly well, even for large rulesets, since the number of partitions is usually small.

The algorithm is sound,^44^ complete,^45^ and generates minimal results.^46^ Consequently, $\mathsf {groupWIs}$ computes an equivalence relation over IP addresses with respect to a simple firewall ruleset for a fixed service. Hence, we call the members of the output set of $\mathsf {groupWIs}$ equivalence classes. Any IP address is a representative for its equivalence class.

#### Theorem 12

($\mathsf {groupWIs}$ sound and generates minimal results) For any two IPs in any equivalence class of $\mathsf {groupWIs}$, the firewall shows the same behavior for a fixed service.

For any two arbitrary equivalence classes *A* and *B* in $\mathsf {groupWIs}$, if we can find two IPs in *A* and *B* respectively where the firewall shows the same behavior for a fixed service, then $A = B$.

*Improving Performance.* We assume that the ruleset has a default policy. Otherwise, we fall back to our previous, slower algorithm. Any simplified, well-formed iptables ruleset has a default policy though.^47^ The above algorithm performs calls to  for each pair of representatives in the partition. The algorithm is significantly slowed down by the quadratic number of calls to . Instead of repeatedly executing  for all combinations of representatives as source and destination address, for a fixed service and fixed source address, we can pre-compute the set of all matching destination addresses with one iteration over the ruleset. The same holds for the matching source addresses. As a rough estimate, this brings down the quadratic number of calls to  to a linear number of iterations over the ruleset. Note that the asymptotic runtime is still quadratic. We have implemented this improved algorithm and proven that Theorems 12 and 13 still hold for it. The empirical evaluation shows that this improvement yields a tenfold speedup.

**Fig. 12:**
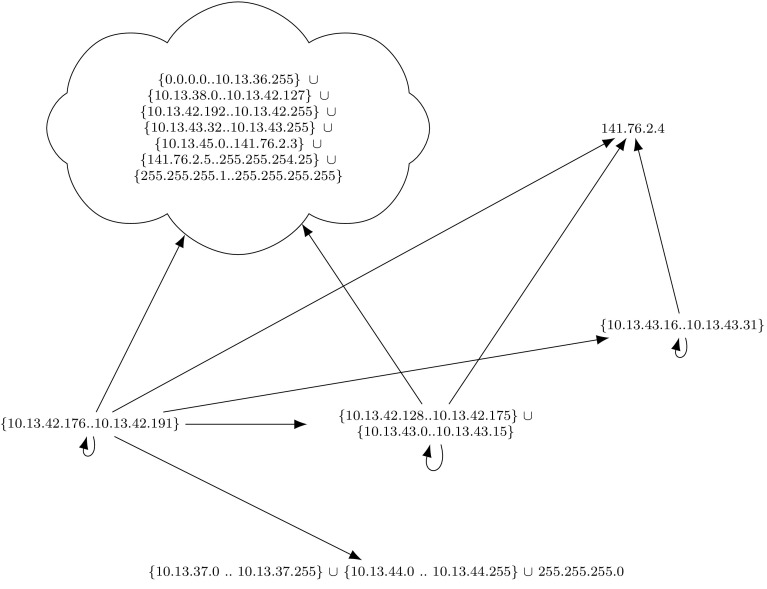
Firewall D SSH service matrix with input and output port rewriting

*Final Theorem.* A service matrix is a square matrix where the number of rows (resp. columns) corresponds to the number of equivalence classes computed by $\mathsf {groupWIs}$. An entry $m_{i,j}$ in a service matrix should mean that all IP addresses in equivalence class *i* are allowed to communicate with all IP addresses in equivalence class *j*. This matrix may not be symmetric and it is not the same as the internal representation used in $\mathsf {groupWIs}$. So far, Theorem 12 only gives guarantees about the layout of the matrix (i.e., rows and columns), but it does not guarantee that the content of the matrix (i.e., the permissions $m_{i,j}$) has the desired property. In addition, we don’t want to present a matrix, but we want to visualize the allowed accesses as graph, for example as shown in e.g., Figs. 7, 8, 9, 10, 11, or 12. Since a service matrix is a square matrix, it can be visualized as graph by treating it as an adjacency matrix. In this way, the function $\mathsf {groupWIs}$ only computes the nodes of the graph.

To draw a graph, for example with TikZ [77] or Graphviz,^48^ one first needs to print the nodes and print the edges afterwards. The name of the nodes (representatives) should not be printed but the IP range they actually represent (equivalence classes). For example, the source code for Fig. 7 may be defined as follows:
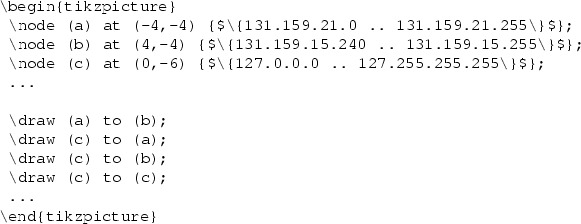


In this example, the node names a, b, and c are identifiers which semantically correspond to the set of IP addresses described by their label. For example, a represents the equivalence class with the range from 131.159.21.0 to 131.159.21.255. The coordinates, for example (-4,-4) for node a are not relevant for our concerns. The edges mean that the complete IP ranges referenced by their representatives may communicate, e.g., 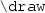 (a) to (b) means that the complete set 131.159.21.0/24 may establish connections to 131.159.15.240/28. In the final drawing, the identifiers a, b, and c are not shown but only their corresponding IP ranges.

A graph $(V,\,E)$ consists of a set of vertices *V* and a set of edges $E \subseteq V \times V$. In our scenario, we have a map $\hat{V}$ where the keys are identifiers (a, b, c, ...) which map to their equivalence class (set of IP addresses). We chose *V* to be the domain of $\hat{V}$. Conveniently, the union of the range of $\hat{V}$ is the universe. We compute the keys of $\hat{V}$ by calling $\mathsf {groupWIs}$ and selecting a representative for each equivalence class (e.g., by taking the lowest IP address). We compute *E* by calling  for each pair of $V \times V$. Note that *V* is minimized and the empirical evaluation shows that this quadratic number of calls to  is not a performance problem. For convenience, we printed symbolic identifiers a, b, c, ...for the keys of $\hat{V}$ instead of IP addresses. We present a final theorem which justifies the correctness of graphs which are drawn according to our method.^49^

#### Theorem 13

(Service Matrix) Let $(\hat{V},\,E)$ be a service matrix. Then, 
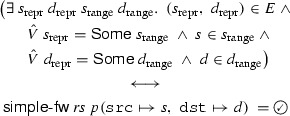


The theorem reads as follows: for a fixed connection, one can look up IP addresses (source *s* and destination *d* pairs) in the graph if and only if the firewall accepts this (*s*, *d*) IP address pair for the fixed connection.

The part which complicates the formalization is the notion of “looking up IP addresses in the graph”. To look up a source IP address *s* in the graph, one first locates *s* as a member in one of the IP equivalence classes, here $s_\mathrm {range}$. This equivalence class is represented by a representative $s_\mathrm {repr}$. The same is done to obtain $d_\mathrm {repr}$. The theorem now says that $(s_\mathrm {repr},\; d_\mathrm {repr}) \in E$ if and only if the firewall allows packets from *s* to *d*. The if-and-only-if relationship in combination with the existential quantifier also implies that there is always exactly one equivalence class in which we can find *s* and *d*, which means that our graph always contains a complete and disjoint representation of the IP address space.

## Stand-Alone Haskell Tool *fffuu*

We used Isabelle’s code generation features [34, 35] to build a stand-alone tool in Haskell. Since all analysis and transformation algorithms are written in Isabelle, we only needed to add parsers and user interface. Overall, more than 80% of the code is generated by Isabelle, which gives us strong trust in the tool.

We call our tool *fffuu*, the “*f*ancy *f*ormal *f*irewall *u*niversal *u*nderstander”.

*fffuu* requires only one parameter to run, namely, an iptables-save dump. This makes it very usable. Optionally, one may pass an *ipassmt*, change the table or chain which is loaded, pass a routing table for output port rewriting, or select the services for the service matrix.

*fffuu* can be easily compiled from source using stack,^50^ which ensures reproducible builds well into the future.

*Example.* We demonstrate *fffuu* by a small example. We want to infer the intention behind the ruleset shown in Fig. 6. Though this ruleset was artificially crafted to demonstrate certain corner cases, it is based on actual rules from real-world firewalls [3, 16]. Also note that the interface name 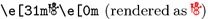 with UTF-8 symbols and shell escapes for color [53] is perfectly valid.

It is hard to guess what the ruleset is implementing. We load the ruleset into *fffuu*, not requiring any additional parameters or manual steps to compute it. The resulting service matrix (for arbitrary ports) is shown in Fig. 7 and provides insight into the intention of the ruleset. An arrow from one IP range to another IP range indicates that the first range may set up connections with the second.

At the bottom, we see the localhost range of 127.0.0.0/8. The reflexive arrow (localhost to localhost) shows that the firewall does not block its own localhost traffic, which is usually a good sign. However, localhost traffic is usually not interesting for a firewall analysis since this range is usually not routed [15]. We will ignore it from now.

On the top, in the cloud, we see a large set of IP addresses. This corresponds to the Internet. On the left, we see the 131.159.21.0/24 range. It may access the Internet and the 131.159.15.240/28 range. On the right, we see the 131.159.15.240/28 range, which may only access the Internet, but not the 131.159.21.0/24 range.

Carefully looking at the figure, we might recognize the overall architecture: the firewall implements the “Demilitarized Zone” (DMZ) architectural pattern. This can usually be described as a local network that is segmented into two parts; a public one that is reachable from the outside Internet (hosting services that need to be reachable from the outside, e.g., a mail or a web server) and an internal one that can only connect to the Internet, but not the opposite direction. To mitigate a situation where some host in the public segment gets compromised, the firewall also prohibits connection from the public into the internal segment. Starting from the original iptables-save input, without the help of *fffuu*, this architecture would have been difficult to uncover and verify.

## Evaluation

We obtained real-world rulesets from over 15 firewalls. Some are central, production-critical devices. They are written by different authors, utilize a vast amount of different features and exhibit different styles and patterns. The fact that we publish the complete rulesets is an important contribution (cf. Wool [82, 84]). To the best of our knowledge, this is the largest, publicly available collection of real-world iptables rulesets. Note: some administrators wish to remain anonymous so we replaced their public IP addresses with public IP ranges of our institute, preserving all IP subset relationships.

Table 1 summarizes the evaluation’s results. The first column (“Fw”) labels the analyzed ruleset. Column 2 (“Rules”) contains the number of rules (only the filter table) in the output of iptables-save. We work directly on these real-world data sets. Column 3 describes the analyzed chain. Depending on the type of firewall, we either analyzed the FORWARD (“FW”) or the INPUT (“IN”) chain. For a host firewall, we analyzed IN; for a network firewall, e.g., on a gateway or router, we analyzed FW. In parentheses, we wrote the number of rules after unfolding the analyzed chain. The unfolding also features some generic, straight-forward optimizations, such as removing rules where the match expression is $\lnot \,{\mathsf {Any}}{}$. Column 4 (“Simple rules”) is the number of rules when translated to the simple firewall. In parentheses, we wrote the number of simple firewall rules when interfaces are removed. This ruleset is used subsequently to compute the partitions and service matrices. In column 5 (“Use”), we mark whether the translated simple firewall is useful. We will detail on the metric later. Column 6 (“Parts”) lists the number of IP address space partitions. For comparison, we give the number of partitions computed by ITVal in parentheses. In Columns 7 and 8, we give the number of partitions for the service matrices for SSH and HTTP. In column 9 (“Time (ITVal)”), for comparison, we put the runtime of the partitioning by ITVal in parentheses in seconds, minutes, or hours. In column 10 (“Time (this)”), we give the overall runtime of our analysis.

When translating to the simple firewall, to accomplish support for arbitrary matching primitives, some approximations need to be performed. For every firewall, the first row states the overapproximation (more permissive), the second row the underapproximation (more strict).

In contrast to the intermediate evaluation, there is no longer the need to manually exclude certain rules from the analysis (cf. Sect. 6.4). For some rulesets, we do not know the interface configuration. For others, there were zone-spanning interfaces. For these reasons, as proven in Sect. 11.6, in the majority of cases, we could not rewrite interfaces. This is one reason for the differences between over- and underapproximation.

We loaded all translated simple firewall rulesets (without interfaces) with iptables-restore. This validates that our results are well-formed. We then used iptables directly to generate the firewall format required by ITVal (iptables -L -n). Our translation to the simple firewall is required because ITVal cannot understand the original complex rulesets and produces flawed results for them.

*Performance.* We have two possibilities to execute our algorithms, depending on whether the user wants to run them inside of Isabelle or as an external stand-alone application [34].

For our evaluation, we utilize Isabelle’s code reflection capabilities. In essence, it gives us a way to execute our algorithms as if they were implemented in Isabelle’s implementation language (Standard ML). Isabelle’s code generator introduces its own unoptimized version for data structures that are already present in the standard libraries of many programming languages. Hence, the generated code may be quite inefficient.^51^ For example, lookups in Isabelle-generated dictionaries have linear lookup time, compared to constant lookup time of standard library implementations. In contrast, ITVal is highly optimized C++ code. We benchmarked our tool on a commodity i7-2620M laptop with 2 physical cores and 8 GB of RAM. In contrast, we executed ITVal on a server with 16 physical Xeon E5-2650 cores and 128 GB RAM. The runtime measured for our tool is the complete translation to the two simple firewalls, computation of partitions, and the two service matrices. In contrast, the runtime of ITVal only consists of computing one partition. The reported time of our tool also includes the runtime of Isabelle’s code generator, but for ITVal we did not add its compilation time. This is one reason why ITVal outperforms our tool for runtimes of < 1 min.

These benchmark settings are biased against our tool. Indeed, exporting our tool to a standalone Haskell application instead, replacing some common data structures with optimized ones from the Haskell standard library, enabling aggressive compiler optimization and parallelization, not counting compilation time, and running our tool on the Xeon server, the runtime of our tool improves by orders of magnitude. Our stand-alone tool *fffuu* also achieves a better runtime by orders of magnitude. Nevertheless, we chose the “unfair” setting to demonstrate the feasibility of running verified code directly in a theorem prover.

Table 1 shows that our tool outperforms ITVal for large firewalls. We added ITVal’s memory requirements to the table if they exceeded 20 GB. ITVal requires an infeasible amount of memory for larger rulesets while our tool can finish on commodity hardware. The overall numbers show that the runtime for our tool is sufficient for static, offline analysis, even for large real-word rulesets.

For our daily use and convenience, we use our Haskell tool *fffuu* which adds another order of magnitude of speedup to our numbers of Table 1.

*Quality of results.* The main goal of ITVal is to compute a minimal partitioning while ours may not be minimal. A smaller number of partitions is better, since the result is more overseeable. It can be seen that ITVal provides better results than our approach in Column 6. Since a service matrix is more specific than a partitioning, the partitions of a service matrix (Column 7 and 8) can be even smaller. Our service matrices are provably minimal and thus improve on ITVal’s partitioning. Since a partitioning cannot be smaller than a service matrix, the numbers in Column 6 must be greater or equal than the numbers in Column 7 or 8. For firewalls *A* and *R*, it can be seen that ITVals’s results are spurious, while ours are provably correct. In general, if the number of partitions calculated by ITVal is smaller than those of a service matrix, this is an error in ITVal.

In Column 5, we show the usefulness of the translated simple firewall (including interfaces). We deem a firewall useful if interesting information was preserved by the approximation. Therefore, we manually inspected the rulesest and compared it to the original. For the overapproximation, we focused on preserved (non-shadowed) ${\mathtt {Drop}}$ rules. For the underapproximation, we focused on preserved (non-shadowed) ${\mathtt {Accept}}$ rules. If the firewall features some rate-limiting for all packets in the beginning, the underapproximation is naturally a drop-all ruleset because the rate-limiting could apply to all packets. According to our metric, such a ruleset is of no use (but the only sound solution). We indicate this case with a superscript ^r^. The table indicates that, usually, at least one approximation per firewall is useful.

For brevity, we only elaborate on the most interesting rulesets and consequences of their analysis.

*Firewall A.* This firewall is the core firewall of our lab (Chair of Network Architectures and Services). It has two uplinks, interconnects several VLANs, and matches on more than 20 interfaces. It has around 500 direct users and one transfer network for an autonomous system (AS) behind it. The traffic is usually several Mbit/s. We have analyzed dumps from Oct 2013, Sep 2014, May 2015, and Sep 2015. The changing number of rules indicates that it is actively managed.

The firewall starts with some rate-limiting rules. Therefore, its stricter approximation assumes that the rate-limiting always applies and transforms the ruleset into a deny-all ruleset. The more permissive approximation abstracts over this rate-limiting and provides a very good approximation of the original ruleset.

The SSH service matrix is visualized in Fig. 8 and in Fig. 9 with the raw IP addresses. The figure can be read as follows: the vast majority of our IP addresses are grouped into *internal* and *servers*. Servers are reachable from the outside, internal hosts are not. $$ ip _1$$ and $$ ip _2$$ are two individual IP addresses with special exceptions. There is also a group for the backbone routers of the connected AS. INET is the set of IP addresses which does not belong to us, basically the Internet. INET’ is another part of the Internet. With the help of the service matrix, the administrator confirmed that the existence of INET’ was an error caused by a stale rule. The misconfiguration has been fixed. Figure 8 summarizes over 4000 firewall rules and helps to easily visually verify the complex SSH setup of our firewall. The administrator was also interested in the Kerberos (kerberos-adm) and LDAP service matrices. They helped verifying the complex setup and discovered potential for ruleset cleanup.

We have used the *fffuu* tool further on to analyze our firewall. For example, Figs. 10 (IPv4) and 11 (IPv6) were created from a recent snapshot of June 2016 and depict the service matrix for HTTP. This snapshot is not listed in the table. The figures show the raw IP addresses. It can be seen that the “two INETs” bug has been fixed, but the overall complexity of the firewall increased. Note that the service matrix is minimal, i.e., there is no way to compress it any further. The two figures reveal the intrinsic complexity of this firewall. However, the figures, though complicated, can still be visualized on one page. This would be impossible for the thousands of rules of the actual ruleset. It demonstrates that our service matrices can give a suitable overview of complicated rulesets.

*Firewall D.* This firewall was taken from a Shorewall system with 373 rules and 65 chains. It can be seen that unfolding increases the number of rules, because of the complex call structures generated by the user-defined chains. Transforming to the simple firewall further increases the ruleset size. This is, among other reasons, due to rewriting several negated IP matches back to non-negated CIDR ranges and NNF normalization. However, the absolute numbers tell us that this blow up is no problem for computerized analysis.

Roughly speaking, the firewall connects interfaces to each other, i.e., it heavily uses -i and -o. This can be easily seen in the overapproximation. There are also many zone-spanning interfaces. As we have proven, it is impossible to rewrite interfaces in this case. In addition, for some interfaces, no IP ranges are specified. Hence, this ruleset is more of a link layer firewall than a network layer firewall. Consequently, the service matrices are barely of any use.

Later on, having obtained more detailed interface and routing configurations, we tried again with input and output port rewriting. The result is not shown in the table, but visualized in Fig. 12. The figure now correctly summarizes the network architecture enforced by the firewall. It shows the general Internet, a Debian update server (141.76.2.4), and four internal networks with different access rights.

*Firewall E.* This ruleset was taken from a NAS device from the introduction (Fig. 1). The ruleset first performs some rate-limiting. Consequently, the underapproximation corresponds to the deny-all ruleset. The table lists a more recent version of the ruleset after a system update. Our SSH service matrix reveals a misconfiguration: SSH was accidentally left enabled after the update. After this discovery, the flaw was fixed. The service matrix for the other services provided by the NAS (not listed in the table) verifies that these services are only accessible from the local network. This finally yields the expected result as motivated in the introduction.

*Firewall F.* This firewall is running on a publicly accessible server. The firewall first allows everything for localhost, then blocks IP addresses which have shown malicious behavior in the past, and finally allows certain services. Since most rules are devoted to blocking malicious IPs, our IP address space partition roughly grows linearly with the number of rules. The service matrices, however, reveal that there are actually only three classes of IP ranges: localhost, the blocked IPs, and all other IPs which are granted access to the services.

*Firewall G.* For this production server, the service matrices verified that a SQL daemon is only accessible from a local network and three explicitly-defined public IP addresses. Our tool could verify the belief of the administrator that the firewall is configured correctly.

*Firewall H.* This ruleset from 2003 appears to block Kazaa filesharing traffic during working hours. In addition, a rule drops all packets with the string “X-Kazaa-User”. The more permissive abstraction correctly tells that the firewall may accept all packets for all IPs (if the above conditions do not hold). Hence, the firewall is essentially abstracted to an allow-all ruleset. According to our metric, this information is not useful. However, in this scenario, this information may reveal an error in the ruleset: the firewall explicitly permits certain IP ranges, but the default policy is ${\mathtt {Accept}}$ and includes all these previously explicitly permitted ranges. By inspecting the structure of the firewall, we suspect that the default policy should be ${\mathtt {Drop}}$. This possible misconfiguration was uncovered by the overapproximation.

The underapproximation does not understand the string match on “X-Kazaa-User” in the beginning and thus corresponds to the deny-all ruleset. However, a manual inspection of the underapproximation still reveals an interesting error: the ruleset also tries to prevent MAC address spoofing for some hard-coded MAC/IP pairs. However, we could not see any drop rules for spoofed MAC addresses in the underapproximation. Indeed, the ruleset allows non-spoofed packets but forgets to drop the spoofed ones. This firewall demonstrates the worst case for our approximations: one set of accepted packets is the universe, the other is the empty set. But because this ruleset is severely broken, no better approximation would be possible. Nevertheless, the manual inspection of the simplified ruleset helped reveal several errors. This demonstrates that even if the service matrices do not contain any information, the other output of our tool may still contain interesting information.

*Firewall P.* This is the ruleset of the main firewall of a medium-sized company. The administrator asked us what their ruleset was doing. They did not reveal their intentions to prevent analysis results skewed towards the expected outcome.

We calculated the simplified firewall rules and service matrices. Using the underapproximation, we could also give guarantees about the packets which are definitely allowed by the firewall. The administrator critically inspected the output of our tool. Finally, they confirmed that the firewall was working exactly as intended. This demonstrates: not only finding errors but showing correctness is one of the key strengths of our tool.

After the analysis, the administrator revealed their true intentions. They have previously upgraded the system to iptables. Their users (the company’s employees) became aware of that. They received some complaints about connectivity issues and the employees were blaming the firewall. However, the administrator was suspecting that the connectivity issues were triggered by some users who are behaving against the corporate policy, e.g., sharing user accounts. With the help of our analysis, the administrator could reject all accusations about their firewall configuration and follow their initial suspicion about misbehaving employees.

A few months later, we received feedback that the firewall was perfect and “users are stupid”.

**Fig. 13:**
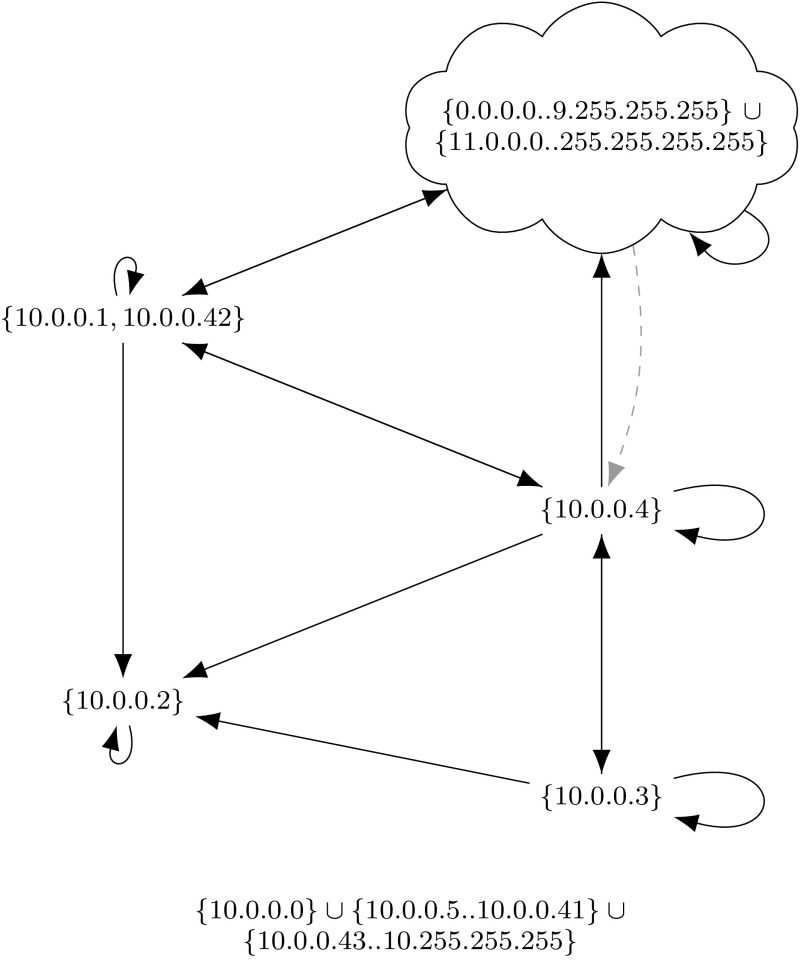
HTTP service matrix with state of a Docker host

*Firewall R.* This ruleset was extracted from a Docker host and partly generated by *topoS* [21]. For remote management, the ruleset allows unconstrained SSH access for all machines, which can be seen by the fact that the SSH service matrix only shows one partition. In contrast, an advanced setup is enforced for HTTP and the HTTP matrix is visualized in Fig. 13. Being able to verify the publicly exposed HTTP setup while neglecting the SSH maintenance setup demonstrates the advantage of calculating our access matrices for each service. We extended *fffuu* to also show flows which can be in an ESTABLISHED state. This is visualized by an orange dashed line. Due to special, scenario-specific requirements, we can see that 10.0.0.2 is a true information sink and may not even answer to ESTABLISHED connections. The lower closure also exhibits one interesting detail: except for one host which is rate limited, SSH connectivity is guaranteed. Ironically, ITVal segfaults on the original ruleset. With our processing, it terminates successfully but returns a spurious result.

## Outlook: Verifying OpenFlow Rules

OpenFlow [64, 67] is a standard for configuring OpenFlow-enabled switches. It is usually referred to in the context of Software-Defined Networking (SDN) and has been hot topic in network management and operations for almost 10 years.

This article focused on the analysis of iptables instead of OpenFlow for several reasons: despite OpenFlow 1.0 [67] having been available for over 5 years, it is a relatively young and not very wide-spread product. In contrast, iptables is battle-tested, real-world approved, supports a large amount of features, and has been in productive use for over a decade. There are also decade-old configurations which utilize a vast amount of features, which are no longer fully understood by administrators [3]. As of July 2016, the popular systems and networking Q&A site *Server Fault*^52^ counts more than a hundred times more questions related to iptables than OpenFlow. The related *Super User*^53^ site counts even a thousand times more questions related to iptables than OpenFlow.

Over the years, iptables has evolved into a system with an enormous amount of (legacy) features. Compared to this, OpenFlow is a tidy piece of technology. But we anticipate to see similar feature creep over the years, considering, e.g., Nicira extensions [61] or attempts to enhance OpenFlow with generic FPGAs to add “exotic functionality” [12]. In a broader context, by extending OpenFlow or one of its proposed stateful, more feature-rich, successors [7], many iptables features have already been reimplemented on top of it [65].

Our declared goal was to provide scientific methods to understand challenging configurations (as observed in iptables) and evaluate our methodology on complex, real-world, legacy-grown systems. The insights we obtained can also be applied to OpenFlow. In particular, a large portion of this article focuses on match conditions, e.g., abstracting over unknowns, optimizing, rewriting, normalizing, or even replacing interfaces by IP addresses. Our work on match conditions can be directly reused in future work within the context of OpenFlow.

However, iptables is not OpenFlow. In particular, the OpenFlow standard defines a vast amount of actions which can be performed for a packet. In contrast, iptables filtering primarily uses the two actions $\mathtt {Accept}$ and $\mathtt {Drop}$. This is because a firewall cleanly separates filtering from other network functions, such as packet rewriting. OpenFlow implementations tend to mix those. We have shown how to deal with unknown match conditions, but unknown actions are an unsolved problem. We discussed what would be required for a full OpenFlow semantics. In particular, a mutable packet model (cf. Sect. 4.2) would be necessary, which our methods do not support. However, there is no technical need for OpenFlow switches to mix packet filtering with other operations. For example, the pipelined OpenFlow Router architecture constructed by Nelson et al. [57, Sect. 3, Fig. 3] clearly separates packet filtering from packet forwarding and rewriting. In general, using pipeline processing as specified in recent OpenFlow standards [64] might be a step forward to separate filtering from forwarding and rewriting. This may also help compilers which produce OpenFlow rules and suffer from a large blow-up which is induced by a cross product over several tables to join rules for different actions into one table [76]. Such a filtering table implemented by OpenFlow rules without unspecified behavior could be analyzed by our presented methods.

In contrast to firewall rules, OpenFlow flow table entries are usually not written by hand, but high-level programming languages (such as NetCore [52], NetKAT [4], or Flowlog [56]) can be used. The overall question arises whether the analysis of low-level OpenFlow rules is necessary, since for example a verified compiler from NetCore to OpenFlow exists [33]. Therefore, the analysis and verification of the high-level programming language may be more interesting than the analysis of generated low-level OpenFlow entries. The Flowlog language was especially designed with built-in verification and analysis in mind [55, 58] and NetKAT was explicitly designed as a Kleene Algebra with Tests (KAT) which is suitable for formal analysis and it also features an automated decision procedure [30].

## Conclusion

This work was motivated by the fact that we could not find any tool which helped us analyze our lab’s and other firewall rulesets. Though much related work about firewall analysis exists, all academic firewall models are too simplistic to be applicable to those real-world rulesets. With the transformations presented in this article, they became processable by existing tools.

We have demonstrated the first fully verified, real-world applicable analysis framework for firewall rulesets. Our tool *fffuu* supports the Linux iptables firewall because it is widely used and well-known for its vast amount of features. It directly works on iptables-save output. We presented an algebra on common match conditions and a method to translate complex conditions to simpler ones. Further match conditions, which are either unknown or cannot be translated, are approximated in a sound fashion. This results in a translation method for complex, real-world rulesets to a simple model. The evaluation demonstrates that, despite possible approximation, the simplified rulesets preserve the interesting aspects of the original ones.

Based on the simplified model, we presented algorithms to partition the IPv4 and IPv6 address space and compute service matrices. This allows summarizing and verifying the firewall in a clear manner.

The analysis is fully implemented in the Isabelle theorem prover. No additional input or knowledge of mathematics is required by the administrator.

The evaluation demonstrates applicability on many real-world rulesets. For this, to the best of our knowledge, we have collected and published the largest collection of real-world iptables rulesets in academia. We demonstrated that our approach can outperform existing tools with regard to correctness, supported match conditions, CPU time, and memory requirements. Our tool helped to verify lack of errors or, alternatively, to discover previously unknown errors in real-world, production rulesets.

## Availability

Our Isabelle/HOL theory files with the formalization and the referenced correctness proofs and our tool *fffuu* are available at

https://github.com/diekmann/Iptables_Semantics

It is the first fully machine-verified iptables analysis tool. A stable version of the theory files can also be obtained from the “Archive of Formal Proofs” (AFP) [19, 22, 24, 51]. AFP maintenance policy ensures that our formalization will keep working with newer Isabelle releases.

The raw data of the analyzed firewall rulesets can be found at

https://github.com/diekmann/net-network

To the best of our knowledge, this is the largest, publicly-available collection of real-world iptables firewall rulesets.

## Footnotes

1 Firewalls can be stateful or stateless. Most current firewalls are stateful, which means the firewall remembers and tracks information of previously seen packets, e.g., the TCP connection a packet belongs to and the state of that connection (“conntrack” in iptables parlance). ITVal does not track the state of connections. Match conditions on connection states are treated exactly the same as matches on a packet header. In general, by focusing on rulesets and not firewall implementation, matching on conntrack states is exactly like matching on any other (stateless) condition. However, internally in the firewall, not only the packet header is consulted but also the current connection tables. Note that existing firewall analysis tools also largely ignore state [54]. In our semantics, we also model stateless matching.

2 Note that the other direction is considered easy [71], because basic Cisco IOS access lists have “no nice features” [32]. Also note that there also are *Advanced* Access Lists.

3 Formalization: inductive iptables_bigstep [19].

4 Formalization: theorem iptables_bigstep_r_eq.

5 Formalization: file Semantics_Stateful.thy [19].

6 Formalization: theorem iptables_bigstep_deterministic [19].

7 Formalization: theorem semantics_bigstep_defined [19].

8 For example, setting $\mathtt {CONNMARK}$ in the filter table where it was not necessary, redirecting packets to userspace with $\mathtt {NFQUEUE}$ where we do not know how the userspace application handles them, or specialized loggings such as $\mathtt {NFLOG}$ which is technically equivalent to the $\mathtt {LOG}$ action and could directly be supported.

9 Formalization: theorem unfolding_n_sound_complete [19].

10 The relevant check is in mark_source_chains, file source/net/ipv4/netfilter/ip_tables.c of the Linux kernel version 4.10.

11 Formalization: theorem iptables_bigstep_rw_Reject [19].

12 Formalization: theorem iptables_bigstep_rm_LogEmpty [19].

13 Formalization: theorem unfold_optimize_ruleset_CHAIN [19].

14 Formalization: theorem unfold_optimize_common_matcher_univ_ruleset_CHAIN [19].

15 The final decision for ${\mathtt {Call}}$ (resp. ${\mathtt {Return}}$) rules depends on the called (resp. calling) chain.

16 Formalization: theorem FinalAllowClosure [19].

17 Formalization: theorem FinalDenyClosure [19].

18 Formalization: theorems transform_remove_unknowns_upper, transform_remove_unknowns_lower [19].

19 We revalidated this observation in September 2014 and found that in our firewall, which has seen more than 15 billion packets (${>}{19 \mathrm{TB}}$ data) since the last reboot, more than 95% of all packets matched the first RELATED,ESTABLISHED rule.

20 The same can be concluded for reflexive ACLs in Cisco’s IOS Firewall [14].

21 Since match expressions do not contain disjunctions, any match expression in NNF is trivially also in *Disjunctive Normal Form* (DNF).

22 Formalization: theorem normalized_nnf_match_normalize_match [19].

23 Formalization: theorem normalize_match_correct [19].

24 Formalization: theorems $\beta _\mathrm {magic}$ _approximating_bigstep_fun_iff_iptables_bigstep, LukassLemma [19].

25 Note that this is not the same function as in Sect. 4, because this simple $\mathsf {smatch}$ function does not require parameter $\gamma$. Roughly speaking, it already has the primitive matcher hard-coded into it.

26 Formalization: theorem empty_match [22].

27 Formalization: theorem simple_match_any [22].

28 Formalization: theorem simple_match_and_correct [22].

29 Formalization: theorem simple_match_and_valid [22].

30 Formalization: theorems rewrite_Goto_chain_safe, unfold_optimize_common_matcher_univ_ruleset_CHAIN [19].

31 Formalization: theorems new_packets_to_simple_firewall_overapproximation, new_packets_to_simple_firewall_underapproximation [19].

32 All lemmas and results of the following subsections ultimately yield Theorem 11 and are referenced in its proof.

33 Formalization: theorem ipcidr_conjunct_correct [24].

34 Formalization: theorem cidr_split_prefix [24].

35 Formalization: file Semantics_Stateful.thy [19].

36 This holds because the semantics does not modify a packet during filtering.

37 As a side note, OpenFlow (technically, the Open vSwitch) defines CIDR-like matching for layer 4 ports. With the small change of converting ports to CIDR-like notation, our simple firewall can be directly converted to OpenFlow and we have the first (almost) fully verified translation of iptables rulesets to SDN [50].

38 Note that this cannot be expressed directly in one rule with iptables. In the example, we used the semantics of $\mathtt {Return}$ to construct a compound negated match expression.

39 We strongly discourage the use of “ip link set eth0 name +” [18].

40 Formalization: theorem iface_replace_needs_ipassmt_disjoint [19].

41 Formalization: theorem rpf_strict_correct [51].

42 Formalization: theorem getParts_samefw [22].

43 Formalization: theorem getParts_complete [22].

44 Formalization: theorem build_ip_partition_same_fw [22].

45 Formalization: theorem build_ip_partition_complete [22].

46 Formalization: theorem build_ip_partition_same_fw_min [22].

47 Since we can easily check at runtime whether a ruleset has a default policy, this fallback solely exists for the purpose of stating our theorems without requiring the assumption of a default policy. Our faster algorithm (with default policy) and slower algorithm (without default policy) compute the same result. In practice, any ruleset has a default policy and the faster algorithm is always used.

48 http://www.graphviz.org/.

49 Formalization: definition access_matrix, theorem access_matrix [22].

50 https://docs.haskellstack.org/en/stable/README/.

51 There are methods to improve the performance and provably preserve correctness [43, 44], which are out of the scope of this article.

52 https://serverfault.com/.

53 https://superuser.com/.

## References

[1] Al-Shaer, E., Alsaleh, M.: ConfigChecker: a tool for comprehensive security configuration analytics. In: Configuration Analytics and Automation (SAFECONFIG), pp. 1–2. IEEE (2011). 10.1109/SafeConfig.2011.6111667

[2] Al-Shaer, E., Hamed, H.: Discovery of policy anomalies in distributed firewalls. In: Annual Joint Conference of the IEEE Computer and Communications Societies (INFOCOM), vol. 4, pp. 2605–2616 (2004). 10.1109/INFCOM.2004.1354680

[3] Analyzed firewall rulesets (raw data). https://github.com/diekmann/net-network. Accompanying material

[4] Anderson, C.J., Foster, N., Guha, A., Jeannin, J.B., Kozen, D., Schlesinger, C., Walker, D.: NetKAT: semantic foundations for networks. In: 41st ACM SIGPLAN-SIGACT Symposium on Principles of Programming Languages, POPL ’14, pp. 113–126. ACM, San Diego (2014). 10.1145/2535838.2535862

[5] Baker, F., Savola, P.: Ingress filtering for multihomed networks. RFC 3704 (Best Current Practice) (2004)

[6] Bartal, Y., Mayer, A., Nissim, K., Wool, A.: Firmato: a novel firewall management toolkit. In: IEEE Symposium on Security and Privacy, pp. 17–31. IEEE (1999). 10.1109/SECPRI.1999.766714

[7] Bianchi G, Bonola M, Capone A, Cascone C (2014). OpenState: programming platform-independent stateful openflow applications inside the switch. ACM SIGCOMM Comput. Commun. Rev..

[8] Brucker, A.D., Brügger, L., Kearney, P., Wolff, B.: Verified firewall policy transformations for test case generation. In: 3rd International Conference on Software Testing, Verification and Validation, pp. 345–354. IEEE (2010). 10.1109/ICST.2010.50

[9] Brucker, A.D., Brügger, L., Wolff, B.: Model-based firewall conformance testing. In: Suzuki, K., Higashino, T., Ulrich, A., Hasegawa, T. (eds.) Testing of Software and Communicating Systems, pp. 103–118. Springer (2008)

[10] Brucker, A.D., Brügger, L., Wolff, B.: Formal firewall conformance testing: an application of test and proof techniques. Softw. Test. Verif. Reliab. (STVR) **25**(1), 34–71 (2015). 10.1002/stvr.1544, https://www.brucker.ch/bibliography/abstract/brucker.ea-formal-fw-testing-2014

[11] Brucker, A.D., Brügger, L., Wolff, B.: Formal network models and their application to firewall policies. Archive of Formal Proofs (2017). http://isa-afp.org/entries/UPF_Firewall.shtml. Formal proof development

[12] Byma, S., Tarafdar, N., Xu, T., Bannazadeh, H., Leon-Garcia, A., Chow, P.: Expanding OpenFlow capabilities with virtualized reconfigurable hardware. In: ACM/SIGDA International Symposium on Field-Programmable Gate Arrays, FPGA ’15, pp. 94–97. ACM, Monterey (2015). 10.1145/2684746.2689086

[13] Capretta, V., Stepien, B., Felty, A., Matwin, S.: Formal correctness of conflict detection for firewalls. In: Workshop on Formal Methods in Security Engineering, pp. 22–30. ACM (2007). 10.1145/1314436.1314440

[14] Cisco IOS firewall—configuring IP access lists. Document ID: 23602 (2007). http://www.cisco.com/c/en/us/support/docs/security/ios-firewall/23602-confaccesslists.html

[15] Cotton, M., Vegoda, L., Bonica, R., Haberman, B.: Special-purpose IP address registries. RFC 6890 (Best Current Practice) (2013)

[16] CrazyCat: iptables multiport and negation. Server Fault Question (2016). http://serverfault.com/questions/793631/iptables-multiport-and-negation/

[17] Deering, S., Hinden, R.: Internet protocol, version 6 (IPv6) specification. RFC 2460 (Draft Standard) (1998). Updated by RFCs 5095, 5722, 5871, 6437, 6564, 6935, 6946, 7045, 7112

[18] Diekmann, C.: Naming network interfaces. In: PoC||GTFO: Pastor Laphroaig Races the Runtime Relinker and Other True Tales of Cleverness and Craft, vol. 16, no. 08, pp. 45–46 (2017)

[19] Diekmann, C., Hupel, L.: Iptables semantics. Archive of Formal Proofs (2016). http://isa-afp.org/entries/Iptables_Semantics.shtml. Formal proof development

[20] Diekmann, C., Hupel, L., Carle, G.: Directed security policies: a stateful network implementation. In: Electronic Proceedings in Theoretical Computer Science on Engineering Safety and Security Systems (ESSS), vol. 150, pp. 20–34. Open Publishing Association, Singapore (2014). 10.4204/EPTCS.150.3

[21] Diekmann, C., Korsten, A., Carle, G.: Demonstrating topoS: theorem-prover-based synthesis of secure network configurations. In: 11th International Conference on Network and Service Management (CNSM), pp. 366–371. Barcelona (2015). 10.1109/CNSM.2015.7367384

[22] Diekmann, C., Michaelis, J., Haslbeck, M.: Simple firewall. Archive of Formal Proofs (2016). http://isa-afp.org/entries/Simple_Firewall.shtml. Formal proof development

[23] Diekmann, C., Michaelis, J., Haslbeck, M., Carle, G.: Verified iptables firewall analysis. In: IFIP Networking 2016. Vienna (2016) 10.1007/s10817-017-9445-1 PMC6044321 30069072

[24] Diekmann, C., Michaelis, J., Hupel, L.: IP addresses. Archive of Formal Proofs (2016). http://isa-afp.org/entries/IP_Addresses.shtml. Formal proof development

[25] Diekmann, C., Posselt, S.A., Niedermayer, H., Kinkelin, H., Hanka, O., Carle, G.: Verifying security policies using host attributes. In: Formal Techniques for Distributed Objects, Components, and Systems: 34th IFIP WG 6.1 International Conference, FORTE, pp. 133–148. Springer, Berlin (2014). 10.1007/978-3-662-43613-4_9

[26] Diekmann, C., Schwaighofer, L., Carle, G.: Certifying spoofing-protection of firewalls. In: 11th International Conference on Network and Service Management (CNSM), pp. 168–172. Barcelona (2015). 10.1109/CNSM.2015.7367354

[27] diekmann/Iptables_Semantics: Issue #113—Port numbers belong to a specific protocol. github (2016). https://github.com/diekmann/Iptables_Semantics/issues/113

[28] Eastep, T.M.: iptables made ease—shorewall (2014). http://shorewall.net/

[29] Engelhardt, J.: Towards the perfect ruleset (2011). http://inai.de/documents/Perfect_Ruleset.pdf

[30] Foster, N., Kozen, D., Milano, M., Silva, A., Thompson, L.: A coalgebraic decision procedure for NetKAT. In: 42nd Annual ACM SIGPLAN-SIGACT Symposium on Principles of Programming Languages, POPL ’15, pp. 343–355. ACM, Mumbai (2015). 10.1145/2676726.2677011

[31] Fuller, V., Li, T.: Classless inter-domain routing (CIDR): the internet address assignment and aggregation plan. RFC 4632 (Best Current Practice) (2006)

[32] Gartenmeister, M.: Iptables vs. Cisco PIX (2005). http://lists.netfilter.org/pipermail/netfilter/2005-April/059714.html

[33] Guha, A., Reitblatt, M., Foster, N.: Machine-verified network controllers. In: 34th ACM SIGPLAN Conference on Programming Language Design and Implementation, PLDI ’13, pp. 483–494. ACM, Seattle (2013). 10.1145/2462156.2462178

[34] Haftmann, F., Bulwahn, L.: Code generation from Isabelle/HOL theories (2016)

[35] Haftmann, F., Nipkow, T.: Code generation via higher-order rewrite systems. In: Blume, M., Kobayashi, N., Vidal, G. (eds.) Functional and Logic Programming, pp. 103–117. Springer (2010)

[36] Hamed H, Al-Shaer E (2006). Taxonomy of conflicts in network security policies. IEEE Commun. Mag..

[37] Hewlett Packard: IP firewall configuration guide (2005). ftp://ftp.hp.com/pub/networking/software/ProCurve-SR-IP-Firewall-Config-Guide.pdf

[38] IPTables Example Config. http://networking.ringofsaturn.com/Unix/iptables.php. Retrieved Sept 2014

[39] Jeffrey, A., Samak, T.: Model checking firewall policy configurations. In: Policies for Distributed Systems and Networks, pp. 60–67. IEEE (2009). 10.1109/POLICY.2009.32

[40] Kawamura, S., Kawashima, M.: A recommendation for IPv6 address text representation. RFC 5952 (Proposed Standard) (2010)

[41] Kazemian, P., Varghese, G., McKeown, N.: Header space analysis: static checking for networks. In: 9th USENIX Symposium on Networked Systems Design and Implementation (NSDI), NSDI’12, pp. 113–126. USENIX Association, San Jose (2012)

[42] Kleene, S.C.: Introduction to Metamathematics. Bibliotheca Mathematica. North-Holland, Amsterdam (1952)

[43] Lammich P (2013). Automatic Data Refinement.

[44] Lammich P, Tuerk T (2012). Applying Data Refinement for Monadic Programs to Hopcroft’s Algorithm.

[45] Leblond, E.: Why you will love nftables (2014). https://home.regit.org/2014/01/why-you-will-love-nftables/

[46] Linux Kernel Sources: Linux/include/linux/netfilter/x_tables.h. Kernel 4.6. http://lxr.free-electrons.com/source/include/linux/netfilter/x_tables.h?v=4.6#L343

[47] Mansmann, F., Göbel, T., Cheswick, W.: Visual analysis of complex firewall configurations. In: 9th International Symposium on Visualization for Cyber Security, VizSec ’12, pp. 1–8. ACM (2012). 10.1145/2379690.2379691

[48] Marmorstein, R.M., Kearns, P.: A tool for automated iptables firewall analysis. In: USENIX Annual Technical Conference, FREENIX Track, pp. 71–81. USENIX Association (2005)

[49] Marmorstein, R.M., Kearns, P., et al.: Firewall analysis with policy-based host classification. In: 20th USENIX Large Installation System Administration Conference (LISA), vol. 6. USENIX Association, Washington (2006)

[50] Michaelis, J., Diekmann, C.: LOFT—verified migration of Linux firewalls to SDN. Archive of Formal Proofs (2016). http://isa-afp.org/entries/LOFT.shtml. Formal proof development

[51] Michaelis, J., Diekmann, C.: Routing. Archive of Formal Proofs (2016). http://isa-afp.org/entries/Routing.shtml. Formal proof development

[52] Monsanto, C., Foster, N., Harrison, R., Walker, D.: A compiler and run-time system for network programming languages. In: 39th Annual ACM SIGPLAN-SIGACT Symposium on Principles of Programming Languages, POPL ’12, pp. 217–230. ACM (2012)

[53] Moy, E., Gildea, S., Dickey, T.: XTerm control sequences (2016). http://invisible-island.net/xterm/ctlseqs/ctlseqs.html

[54] Nelson, T., Barratt, C., Dougherty, D.J., Fisler, K., Krishnamurthi, S.: The Margrave tool for firewall analysis. In: 24th USENIX Large Installation System Administration Conference (LISA). USENIX Association, San Jose (2010)

[55] Nelson T, Ferguson AD, Krishnamurthi S (2015). Static Differential Program Analysis for Software-Defined Networks.

[56] Nelson, T., Ferguson, A.D., Scheer, M.J., Krishnamurthi, S.: Tierless programming and reasoning for software-defined networks. In: 11th USENIX Symposium on Networked Systems Design and Implementation (NSDI), NSDI’14, pp. 519–531. USENIX Association, Seattle (2014)

[57] Nelson, T., Ferguson, A.D., Yu, D., Fonseca, R., Krishnamurthi, S.: Exodus: toward automatic migration of enterprise network configurations to SDNs. In: 1st ACM SIGCOMM Symposium on Software Defined Networking Research, no. 13 in SOSR ’15, pp. 13:1–13:7. ACM, Santa Clara (2015). 10.1145/2774993.2774997

[58] Nelson, T., Guha, A., Dougherty, D.J., Fisler, K., Krishnamurthi, S.: A balance of power: expressive, analyzable controller programming. In: Second ACM SIGCOMM Workshop on Hot Topics in Software Defined Networking, HotSDN ’13, pp. 79–84. ACM, Hong Kong (2013). 10.1145/2491185.2491201

[59] NetCitadel, Inc.: FirewallBuilder. http://www.fwbuilder.org. Ver. 5.1

[60] netfilter coreteam: libxtables/xtables.c. https://git.netfilter.org/iptables/tree/libxtables/xtables.c?h=v1.6.0#n518

[61] Nicira, Inc.: Nicira extensions. openvswitch/ovs repository (2016). https://github.com/openvswitch/ovs/blob/master/include/openflow/nicira-ext.h. Revision fb8f22c186b89cd36059c37908f940a1aa5e1569

[62] Nipkow T, Klein G (2014). Concrete Semantics.

[63] Nipkow, T., Paulson, L.C., Wenzel, M.: Isabelle/HOL: a proof assistant for higher-order logic. In: LNCS, vol. 2283. Springer (2002, last updated 2016). http://isabelle.in.tum.de/

[64] Nygren, A., Pfaff, B., Lantz, B., Heller, B., Barker, C., Beckmann, C., Cohn, D., Malek, D., Talayco, D., Erickson, D., McDysan, D., Ward, D., Crabbe, E., Schneider, F., Gibb, G., Appenzeller, G., Tourrilhes, J., Tonsing, J., Pettit, J., Yap, K., Poutievski, L., Dunbar, L., Vicisano, L., Casado, M., Takahashi, M., Kobayashi, M., Orr, M., Yadav, N., McKeown, N., dHeureuse, N., Balland, P., Madabushi, R., Ramanathan, R., Price, R., Sherwood, R., Das, S., Gandham, S., Curtis, S., Natarajan, S., Mizrahi, T., Yabe, T., Ding, W., Yiakoumis, Y., Moses, Y., Kis, Z.L.: OpenFlow switch specification v1.5.1 (2015). https://www.opennetworking.org/images/stories/downloads/sdn-resources/onf-specifications/openflow/openflow-switch-v1.5.1.pdf.ONFTS-025

[65] Petrucci, L., Bonelli, N., Bonola, M., Procissi, G., Cascone, C., Sanvito, D., Pontarelli, S., Bianchi, G., Bifulco, R.: Towards a stateful forwarding abstraction to implement scalable network functions in software and hardware. ArXiv e-prints (2016)

[66] PF: the OpenBSD packet filter. http://www.openbsd.org/faq/pf/

[67] Pfaff, B., Heller, B., Talayco, D., Erickson, D., Gibb, G., Appenzeller, G., Tourrilhes, J., Pettit, J., Yap, K., Casado, M., Kobayashi, M., McKeown, N., Balland, P., Price, R., Sherwood, R., Yiakoumis, Y.: OpenFlow switch specification v1.0.0 (2009). http://archive.openflow.org/documents/openflow-spec-v1.0.0.pdf

[68] Postel, J.: Internet protocol. RFC 791 (INTERNET STANDARD) (1981). Updated by RFCs 1349, 2474, 6864

[69] Pozo, S., Ceballos, R., Gasca, R.M.: CSP-based firewall rule set diagnosis using security policies. In: 2nd International Conference on Availability, Reliability and Security (ARES), pp. 723–729. IEEE, Los Alamitos (2007). 10.1109/ARES.2007.63

[70] Pozo S, Ceballos R, Gasca RM (2009). Model-based development of firewall rule sets: diagnosing model inconsistencies. Inf. Softw. Technol..

[71] Renard, B.: cisco-acl-to-iptables (2013). http://git.zionetrix.net/?a=summary&p=cisco-acl-to-iptables. Retrieved Sept 2014

[72] Reynolds, J.: Assigned numbers: RFC 1700 is replaced by an on-line database. RFC 3232 (Informational) (2002)

[73] Reynolds, J., Postel, J.: Assigned numbers. RFC 1700 (Historic) (1994). Obsoleted by RFC 3232

[74] Sherry J, Hasan S, Scott C, Krishnamurthy A, Ratnasamy S, Sekar V (2012). Making middleboxes someone elses problem: network processing as a cloud service. ACM SIGCOMM Comput. Commun. Rev..

[75] Sluizer, S., Postel, J.: Mail transfer protocol. RFC 780 (1981). Obsoleted by RFC 788

[76] Smolka, S., Eliopoulos, S.A., Foster, N., Guha, A.: A fast compiler for NetKAT. In: International Conference on Functional Programming (ICFP), pp. 328–341. ACM (2015). 10.1145/2784731.2784761

[77] Tantau, T., Feuersaenger, C.: The TikZ and pgf packages (2016). Pgfversion 3.0.1a

[78] The netfilter.org project: netfilter/iptables project. http://www.netfilter.org/

[79] The netfilter.org project: netfilter/nftables project. http://www.netfilter.org/

[80] Tongaonkar, A., Inamdar, N., Sekar, R.: Inferring higher level policies from firewall rules. In: 21st USENIX Large Installation System Administration Conference (LISA), vol. 7, pp. 1–10. USENIX Association, Dallas (2007)

[81] Verizon Business RISK Team, United States Secret Service: 2010 Data Breach Investigations Report (2010). http://www.verizonenterprise.com/resources/reports/rp_2010-DBIR-combined-reports_en_xg.pdf

[82] Wool A (2004). A quantitative study of firewall configuration errors. IEEE Comput..

[83] Wool A (2004). The use and usability of direction-based filtering in firewalls. Comput. Secur..

[84] Wool A (2010). Trends in firewall configuration errors: measuring the holes in swiss cheese. IEEE Internet Comput..

[85] Yuan, L., Chen, H., Mai, J., Chuah, C.N., Su, Z., Mohapatra, P.: FIREMAN: a toolkit for firewall modeling and analysis. In: IEEE Symposium on Security and Privacy, pp. 199–213 (2006)

[86] Zhang, B., Al-Shaer, E., Jagadeesan, R., Riely, J., Pitcher, C.: Specifications of a high-level conflict-free firewall policy language for multi-domain networks. In: 12th ACM Symposium on Access Control Models and Technologies, SACMAT’07, pp. 185–194. ACM (2007). 10.1145/1266840.1266871

[87] Zhang, S., Mahmoud, A., Malik, S., Narain, S.: Verification and synthesis of firewalls using SAT and QBF. In: Network Protocols (ICNP), pp. 1–6 (2012). 10.1109/ICNP.2012.6459944

